# Targeting Abnormal Hematopoietic Stem Cells in Chronic Myeloid Leukemia and Philadelphia Chromosome-Negative Classical Myeloproliferative Neoplasms

**DOI:** 10.3390/ijms22020659

**Published:** 2021-01-11

**Authors:** Yammy Yung, Emily Lee, Hiu-Tung Chu, Pui-Kwan Yip, Harinder Gill

**Affiliations:** Division of Haematology, Medical Oncology and Haemopoietic Stem Cell Transplantation, Department of Medicine, LKS Faculty of Medicine, The University of Hong Kong, Hong Kong, China; u3558354@hku.hk (Y.Y.); emilylmy@hku.hk (E.L.); u3557654@hku.hk (H.-T.C.); u3557642@hku.hk (P.-K.Y.)

**Keywords:** stem cells, chronic myeloid leukemia, myeloproliferative neoplasm, targeted therapy

## Abstract

Myeloproliferative neoplasms (MPNs) are unique hematopoietic stem cell disorders sharing mutations that constitutively activate the signal-transduction pathways involved in haematopoiesis. They are characterized by stem cell-derived clonal myeloproliferation. The key MPNs comprise chronic myeloid leukemia (CML), polycythemia vera (PV), essential thrombocythemia (ET), and primary myelofibrosis (PMF). CML is defined by the presence of the Philadelphia (Ph) chromosome and *BCR-ABL1* fusion gene. Despite effective cytoreductive agents and targeted therapy, complete CML/MPN stem cell eradication is rarely achieved. In this review article, we discuss the novel agents and combination therapy that can potentially abnormal hematopoietic stem cells in CML and MPNs and the CML/MPN stem cell-sustaining bone marrow microenvironment.

## 1. Introduction

**Figure 1:**
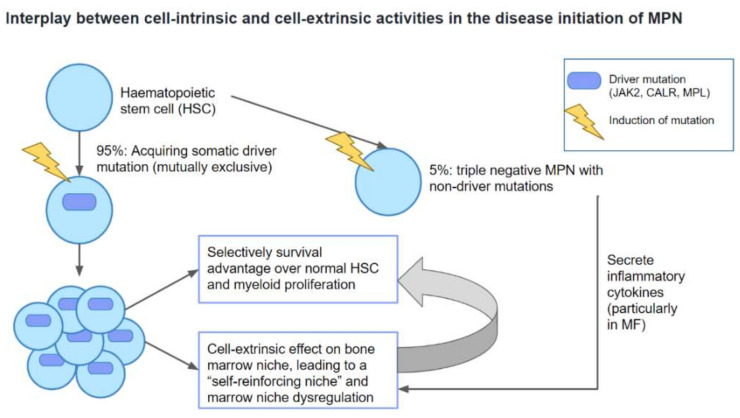
Interplay between cell-intrinsic and cell-extrinsic activities in the disease initiation of MPN. MPN: myeloproliferative neoplasm; MF: myelofibrosis.

**Figure 2:**
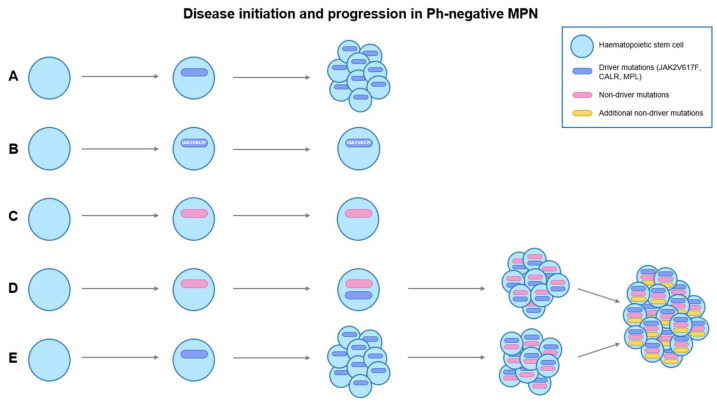
Disease initiation and progression in Ph-negative MPNs. (**A**) shows the acquisition of somatic driver mutations (*JAK2V617F, CALR, MPL*) in a haematopoietic stem cell (HSC), leading to myeloproliferation. (**B**) shows the acquisition of somatic driver mutation, *JAK2*V617F, in HSC without progression to MPN. One of the reasons is the insufficient *JAK2*V617F allele burden to give rise to a MPN phenotype. (**C**) shows the acquisition of non-driver mutations without progression to MPN, indicating the presence of clonal hematopoiesis of indeterminate potential (CHIP). (**D**) The presence of CHIP increases the chance of HSC to acquire driver mutations, leading to myeloproliferation. (**D**,**E**) Acquisition of additional non-driver mutations in MPN stem cells could lead to disease progression or leukemic transformation to secondary acute myeloid leukemia (sAML).

Myeloproliferative neoplasms (MPN) are a collection of clonal hematopoietic stem cell disorders characterized by the proliferation of one of more of the hematopoietic lineages [1,2,3,4]. The major clinicopathologic entities comprise chronic myeloid leukemia (CML), polycythemia vera (PV), essential thrombocythemia (ET), and primary myelofibrosis (PMF) [5,6]. CML is defined by the presence of the Philadelphia (Ph) chromosome that results from t(9;22) (q34.1;q11.2) and formation of the constitutively expressed oncoprotein *BCR-ABL1* [7,8]. Philadelphia chromosome-negative myeloproliferative neoplasms (Ph-negative MPNs) arise from a single clonal hematopoietic stem cell (HSC) leading to proliferation of more than one cell lineage, with transitional forms from one entity to another. Over 95% of PV, ET, and PMF are associated with mutually exclusive somatic driver mutations *JAK2*V617F, calreticulin (*CALR*), and myeloproliferative leukemia protein (*MPL*) [9,10,11,12,13]. Acquisition of somatic driver mutations leads to the development of the MPN stem cells. In addition, disease initiation and progression of Ph-negative MPNs involve the interplay between cell-intrinsic and cell-extrinsic activities. This is characterized by survival advantage of MPN stem cells over normal HSCs that is sustained by a dysregulated bone marrow niche via a positive feedback mechanism [9,14] (Figure 1). Further acquisition of non-driver mutations then plays a pivotal role in determining disease phenotype and promoting leukemic progression [9,10,13] (Figure 2). At diagnosis, all CML patients harbor abnormal HSCs [8,15,16]. They are characterized by an unlimited potential and unrestricted ability to self-regenerate, remain quiescent, mediate *BCR-ABL1*-independent tyrosine kinase inhibitor (TKI)-resistance and evade the host immunity, and allowing disease initiation, development, maintenance and progression [8,15,17,18,19,20,21,22]. The abnormal HSCs in CML are able to survive and thrive through various mechanisms such as modulation of downstream signaling pathways (e.g., JAK/STAT, PI3K/AKT/mTOR, Wnt/β-catenin, Hedgehog signalling), induction of autophagy, selective advantage in homing and engraftment in the bone marrow microenvironment (BMM), and alterations in cellular metabolism [8,15,23,24,25,26,27,28,29]. While leukemia stem cells (LCSs) are generally CD34^+^/CD38^−^, the abnormal HSC populations in CML have extremely heterogeneous and unstable cell surface antigens expression, and vary greatly in terms of their leukemogenic capacity [8,17,21,30,31]. Although TKIs show some efficacy in targeting abnormal HSCs in CML, they may not be adequate for disease eradication.

The persistence of abnormal HSCs in CML and MPN has led to the development of novel therapies targeting CML or MPN stem cells. In this review, we discuss the current and emerging therapeutic options that may target CML and MPN stem cells with the aim of disease modification and eradication. The various pathways involved in the biology of CML and Ph-negative MPNs are depicted in Supplementary Materials Files S2–S7, highlighting the rationale of their therapeutic targeting.

## 2. Current Therapeutic Options in CML and Their Effects on CML Stem Cells

TKIs competitively bind to the ATP-binding site of the BCR-ABL1 to reduce abnormal phosphorylation of the dysregulated tyrosine kinase and inhibit downstream pathways and leukaemogenesis [7,32,33]. TKIs (Table 1) have modest effects on CML stem cells as single agents may be rendered ineffective in targeting CML stem cells as a result of BCR-ABL1-independent mechanisms. They may arise from mutations in epigenetic regulators (e.g., *DNMT3A*, *EZH2*, *IDH1/2*) [7,34] or the loss of tumour suppressor genes (e.g., *TP53*, *PTEN*, *TET1/2*) [17,29] and genes that code for anti-oxidant systems (e.g., FoxO, EPAS1) [17]. Quiescence of CML stem cells is another major challenge, and may contribute to TKI resistance and relapse in CML. Despite the fact that 50% of CML patients achieve treatment-free remissions without relapse after achieving deep molecular response, most harbour residual CML stem cells [15,17,35].

### 2.1. First and Second Generation TKIs

Imatinib (IM), a first generation TKI, is highly effective in inducing apoptosis in *BCR-ABL1*-positive cells. CML stem cells have dysregulated intracellular calcium signalling, uncontrolled pro-inflammatory cytokines such as interleukin-6 (IL-6), IL-8 and nuclear factor kappa beta (NF-κB), and overproduction of activator protein 1 (AP-1), which are hallmarks for CML stem cell survival [29,39,40,41]. In-vivo and in vitro studies showed that these could be reversed by IM antagonizing inositol-triphosphate (IP3)-mediated calcium mobilization (*p* < 0.05) and oxidative stress via IP3 receptor inhibition (IP3R) on the endoplasmic reticulum (ER) [39,40,42]. Herrmann et al. demonstrated a decrease in CD26^+^ stem cells after in vitro IM therapy [16], yet Willmann et al. showed otherwise [36]. Moreover, nilotinib induced CML stem cell apoptosis [36], and nilotinib and dasatinib showed higher potency in IP3R inhibition [42]. IM may also downregulate overexpressed EZH2 in CML stem cells, with minimal effects in normal HSCs [17,43]. Also, in-vitro studies showed that post-dasatinib or -IM therapy, programmed death receptor 1 (PD-1, immune marker for immune-evasion) expression was found to be reduced on CD8^+^ T cells and monocytic myeloid-derived suppressor cells (MDSCs), leading to increased cytotoxic T-lymphocyte- (CTL) and Natural Killer (NK) cell-mediated cytotoxicity [18,44,45,46]. However, contradicting evidence was presented in another in vitro study, which showed that IM enhanced mRNA and protein expression of autophagy-related 4B cysteine peptidase (Atg4B), resulting in TKI-induced autophagy and selective survival in CD34^+^ CML cells (*p* ≤ 0.05) [39].

### 2.2. Ponatinib

Ponatinib, a third generation TKI, is indicated in CML with *BCR-ABL1*^T315I^ mutations or refractoriness to ≥2 TKIs. Steric hindrance is produced due to replacement of threonine by isoleucine at the ATP-binding site [47,48]. Presence of a carbon-carbon triple bond in ponatinib allows it to be 500-fold more potent than IM in overcoming TKI-resistance [22,47,48,49]. Other pathways targeted by ponatinib include VEGFR, KIT, SRC, FGFR, PDGFR, FLT3, and KIT [47]. In-vivo murine models with CML stem cells that are lin^−^Sca-1^+^c-kit^+^ showed that ponatinib was effective in CML stem cell eradication and spleen size reduction. Thirty-percent residual *BCR-ABL1* chimerism at 28 days was achieved compared to >50% in dasatinib and IM [22,47,50].

### 2.3. Asciminib

Asciminib (ABL001), a recent, FDA-approved, fourth generation TKI, is an allosteric inhibitor that binds to the BCR-ABL1 myristoyl-pocket (STAMP) [8,33,49,51,52]. It is effective against *BCR-ABL1* KD-dependent and -independent mutations as monotherapy or in combination with other TKIs to restore TKI-sensitivity in resistant cell lines and produce drug synergism in reducing CRK-like protein (CRKL) phosphorylation for CML stem cells [49,53]. Initial results in a phase I trial (ClinicalTrials.gov number, NCT03595917) demonstrated that 82% of patients with TKI-resistance achieved major cytogenetic response (MCyR) by 3 months and 30% of patients reached CCyR at 5 months [51]. In the phase III ASCEBEL trial, asciminib showed superiority over bosutinib in achieving MMR at 24 weeks [53]. Ongoing trials using asciminib as monotherapy, in combination with other TKIs and/or corticosteroids are underway (ClinicalTrials.gov identifiers: NCT04216563, NCT03906292, NCT04360005, NCT03106779, NCT03595917, NCT03578367 and NCT02081378).

### 2.4. Interferon-α

IFNα was used as first-line treatment before the emergence of TKIs. It induces apoptosis of LSCs via Fas-receptors upregulation, FADD/caspase-8 pathway activation, and cytochrome-c release, leading to mitochondrial disruption and cellular apoptosis independent of anti-apoptotic B-cell lymphoma 2 (Bcl-2), cell-cycle arrest and tumour-suppressor p53 [54,55,56]. IFNα also restores normal function of the dysregulated BMM through β1-integrin for cellular differentiation and elimination of the protective barrier established for LSC quiescence [54,57,58]. IFN-α-mediated increase in expression of major histocompatibility complex (MHC) class I molecules and tumour-associated antigens cause reactivation of CTL and prompt CTL-mediated cytotoxicity against LSCs [54,55]. The 5-year survival rate of IFNα was 57% as shown in a meta-analysis of 7 data sets of randomized trials consisting of 1,554 patients [54,59]. In another study using IFNα monotherapy, the 10-year survival rate was 72%, where 46% remained in CCyR [55,60]. These highlight the potential re-emergence of IFNα for LSC elimination, where clinical trials using IFNα alone or in combination with other TKIs showed promising results for TFR (ClinicalTrials.gov identifiers: NCT02001818, NCT01657604, NCT03117816, NCT03831776, NCT04126681, NCT01316250, NCT02381379, and NCT00452023).

## 3. Current Therapeutic Options in MPN and Their Effects on MPN Stem Cells

### 3.1. IFNα

A major significance of Peg-IFNα-2a is its ability to target MPN stem cells and reduce mutant allele burden in MPN [61,62,63,64,65,66,67,68]. Sustained molecular, haematological response and regression of BM fibrosis were seen in some patients after discontinuation of Peg-IFNα-2a, indicating the eradication of MPN stem cells [65,69] (Table 2). Interestingly, the effect of Peg-IFNα-2a on *JAK2*V617F+ stem cells was greater than that on CALR-mutated stem cells, with no difference in hematological response [70,71]. This is due to the phosphorylation and activation of JAK1-STAT1 pathway in *JAK2*V617F cells, but not in *CALR*-mutated cells, resulting in *JAK2*V617F-positive cells priming towards Peg-IFNα-2a [70]. There is a paucity of data suggesting that Peg-IFNα-2a targeting MPL-mutated clones could be due to the low frequency of *MPL* mutations in MPN.

### 3.2. JAK Inhibitors

Treatment with ruxolitinib showed some reduction in *JAK2*V617F mutant allele burden in PV patients [74,75], although its effect on reduction of mutant allele burden in MF is mild [76]. Additionally, several second generation JAK inhibitors were developed to improve efficacy and reduce side effects of ruxolitinib [77,78,79,80,81]. However, none of them show significance in eradicating LSCs [77,79,80,82,83]. Some studies have shown that fedratinib, a newly FDA approved JAK2/FLT3 inhibitor in 2019, reduced *JAK2*V617F variant allele frequency (VAF) [79,84]. Yet, the results were not consistent with other trials [79,83,85]. Therefore, combinations of various novel therapies with ruxolitinib emerge in the hope of eliminating MPN LSCs [86].

### 3.3. Allogeneic HSCT

Allo-HSCT is able to overcome high molecular risks (HMR) mutations, and its recipients usually harbor additional molecular mutations. These additional molecular mutations, including those conferring a poor prognosis in MPN (*ASXL1*, *EZH2*, *SRSF2*, *IDH1/2*, *TP53*) generally do not affect relapse-free survival (RFS) and OS in patients receiving allo-HSCT [87]. However, discrepancies are seen in terms of relapse risks. The *ASXL1* mutation, which accounts for >90% in intermediate-2 and high-risk MF patients, is found to be associated with higher relapse risks [88]. In a study assessing the outcome of allo-HSCT in MPL-mutated PMF and secondary myelofibrosis (SMF), the only relapsed patient harbored *ASXL1* and *EZH2* mutation [89]. Meanwhile, some post-transplant *ASXL1*-mutated patients may die without relapsing [87].

## 4. Novel Therapy Targeting Signaling Pathways in CML Stem Cells

### 4.1. Novel Tyrosine Kinase Inhibitors

PF-114 is an orally bioavailable fourth generation TKI that is selectively active against *BCR-ABL1*-dependent and -independent mutations, as well as non-mutational TKI-resistant cell lines [8,90,91,92]. It acts as a BCR-ABL1 KD antagonist and/or STAMP inhibitor, suppressing the constitutively active PI3K/AKT/ERK1/2 and JAK/STAT3/5 signalling, elevating p27 levels, leading to G_1_ cell cycle arrest [91,93]. It induced apoptosis in patient-derived K562 and KCL-22 cell lines [91]. In K562 nude mouse xenograft, it caused complete eradication of the tumour bulk (*p* < 0.001) without recurrence [90]. It also showed excellent toxicity profile as it spared VEGFR, FLT3, EPHRIN, FGFR and B-RAF, implying less cardiac, pulmonary and vascular complications [90], hence making it a promising agent for patients refractory and/or resistant to frontline therapies. Phase I/II trials showed that 55% of heavily pretreated patients receiving PF-114 300mg daily achieved MCyR and 36% achieved MMR [92].

### 4.2. Targetting microRNAs

MicroRNAs (miR) are short pleiotropic non-coding RNA sequences that cleave or repress transcription, hence controlling at least 10–40% of human mRNA expression [94,95]. Malfunctioning miR is key to leukemogenesis and TKI-resistance, maintenance and self-regeneration [17,94,95,96]. Overt expression of miR-29a leads to the depletion of tumor suppressor TET2 and antioxidant-coding EPAS1, with upregulation of anti-apoptotic genes Bcl-2 and Mcl-1 apoptosis regulator (Mcl-1) [94,97,98,99]. Downregulation of Bcl-2 inhibitor miR-153-3p is mediated by uncontrolled c-Myc/miR-150 expression in LSC and Ph+ cells, impairing myeloid differentiation and promoting TKI-resistance [100,101]. Prolonged exposure of TKIs triggers drug resistance in LSCs and K562 cells, and is associated with low miR-217 and high DNA methyltransferases (DNMTs) [94,102,103]. miR-424 acts as a direct inhibitor of BCR-ABL1, showing markedly low expression in CD34^+^/CD38^−^ and BCR-ABL1^+^ cells alongside high levels of oncoprotein Cobll1 [94,97,104,105]. In vitro studies suggested that miR-217 overexpression might allow restoration of tumour suppressor effects [94,97,104]. Reduced tumor suppressor miR-142 levels in LSCs and TKI-resistant cells were found to be associated with excessive oncoproteins Mcl-1, cKIT, and SRI, precipitating unimpeded PI3K/AKT, JAK/STAT, and RAS/RAF/MEK/ERK downstream signalling with anti-apoptotic, pro-survival and therapy-resistant effects [94,97,106,107,108,109]. Correction of dysregulated miR levels in preclinical studies exhibited propitious results in reduction of tumour bulk. This may not only yield a new field of clinical investigation for targeted therapy, but also as a tool in aiding diagnosis, a prognostic indicator and a predictor of treatment response [110].

### 4.3. Targeting BCR-ABL1/Gab2/Grb2 Axis

Dysregulated growth factor receptor-bound protein 2 (Grb2) expression permits direct binding to the Src homology (SH2) domain of BCR-ABL1, forming Grb2-SOS complexes and leading to downstream hyperactivation of RAS/MAPK pathway [111,112,113,114]. BP1001, a liposome-incorporated antisense oligodeoxynucleotide targeted against the Grb2 mRNA, inhibits subsequent protein expression and the RAS/MEK/ERK pathway. Preclinical studies showed that Grb2 depletion induced LSC knockdown in CD34^+^ cells, without affecting normal HSCs and STAT inhibition [111,114]. Combination with TKIs overcomes resistance and induces drug synergism [113]. A phase I clinical trial showed that BP1001 enhanced the effects of dasatinib by 2–6-fold and reduced phosphorylation of ERK1/2 and Grb2 levels by >25% in 52% and 49% patients respectively [113]. Trametinib, a MEK inhibitor in combination with imatinib inhibited the MEK/ERK and NF-κB-mediated LSC survival, restoring TKI sensitivity in-vitro and in-vivo [18,115,116].

### 4.4. Targeting MAPK/MNK1/2 Pathway

The MAPK/MNK1/2 pathway is amplified in CML stem cells but not in normal HSCs. The constitutive phosphorylation of eukaryotic initiation factor 4E (eIF4E, an oncoprotein essential for LSC proliferation) induces nuclear activation and translocation of β-catenin, contributing to leukemogenesis and TKI-resistance [117]. Preclinical studies showed that ETC-1907206, a selective MNK1/2 inhibitor, suppressed eIF4E phosphorylation and β-catenin signalling [117].

### 4.5. Targeting mTOR Pathway

mTOR, is a catalytic kinase for the protein complexes mTORC1 and mTORC2 in the PI3K/AKT/mTORC1 pathway [118,119]. It inhibits mRNA translation through initiation factor 4E binding protein (4E-BP1) and p70 ribosomal S6 kinase (p70S6K, S6K) [118,119,120]. Liver kinase B1 (LKB1, tumour suppressor), an upstream kinase of AMP-activated protein kinase (AMPK) induces AMPK phosphorylation of tuberous sclerosis complex 2 (TSC2) to suppress Ras homolog enriched in brain (Rheb) and inhibit mTOR. In LSCs, dysregulated PI3K/AKT/mTOR signalling increases reactive oxygen species (ROS) production, leading to the loss of negative regulation by LKB1 and AMPK, promoting survival, proliferation, drug resistance and stemness [118,121]. Preclinical studies showed that metformin, an AMPK activator inhibited aberrant PI3K/AKT/mTOR hyperactivation to reduce oxidative phosphorylation of glucose and fatty acids, leading to LSC apoptosis [122,123]. However, this led to the compensatory upregulation of glucose and glycolysis which could be be overcome by the addition of 2 deoxy-glucose (2-DG), a hexokinase inhibitor that mimics d-glucose and inhibits glycolysis to induce cell death [123,124]. Combination with TKIs boosts effects and induces TKI-mediated apoptosis [123,124]. Resveratrol, a natural antioxidant found in grapes is also found to stimulate AMPK activation, hence upregulating p38 expression and JNK-mediated phosphorylation of H2AX, downregulating Bcl-2 and triggering caspase-3-mediated LSC apoptosis and cell-cycle arrest [120,125,126]. 5-aminoimidazole-4-carboxamide riboside (AICAR), undergoes phosphorylation and binds to an allosteric site of AMPK to activate it and inhibits mTOR regardless of TKI sensitivity [127].

### 4.6. Bcl-2 Targeting

Bcl-2, a key anti-apoptotic gene that regulates mitochondrial-mediated apoptosis through the JAK/STAT and PI3K/AKT pathways [8,18,128,129], is overwhelmingly expressed in LSCs. In addition, BCR-ABL1 induces upregulation of Bcl-2 anti-apoptotic proteins, including Mcl-1 and B-cell lymphoma-extra large (Bcl-xL) [8,18,128]. Venetoclax, a Bcl-2 inhibitor, binds to the hydrophobic groove of the Bcl-2 homology 3 (BH3) domain of Bcl-2, releasing its inhibition on Bcl-2-associated X protein (BAX) to drive p53/BAX-mediated programmed cell death [18,128]. Preclinical studies showed synergism between venetoclax and TKI in targeting mitochondrial oxidative phosphorylation to eliminate CD34^+^/CD38^+^ and CD34^+^ cells [18,128]. A retrospective study using venetoclax in combination with TKIs showed 60% complete remission (CR), 75% ORR, median survival of 10.9 months and median RSF of 3.9 months [130].

### 4.7. JAK2 Inhibition

JAK2 mediates cytokine-mediated signaling in CML cells. It leads to uncontrolled STAT3/5 phosphorylation by directly binding to the SH2 domain of BCR-ABL1, which is stabilized by Abelson helper integration site 1 (AHI-1), an oncogenic adaptor for LSC survival. [18,30,131,132]. In LSCs, induced expression of MPL enhances JAK/STAT signaling to trigger ROS formation and subsequent clonal evolution, contributing to stemness and TKI-resistance [8,18,30,131]. However, LSCs remain sensitive to JAK2 inhibitors such as ruxolitinib. Preclinical studies showed that combining ruxolitinib with the CML-specific TKIs eliminated CD34^+^/CD38^−^ stem cells with no effects on normal HSCs in-vitro, and reduced CD34^+^ cell engraftment to the BM in-vivo [131]. Sweet et al. showed that 33% of patients had ≥1 log reduction in *BCR-ABL1* transcripts and 44% achieved MR4.5 when co-treated with nilotinib in a phase I trial [133]. Another phase I trial with nilotinib demonstrated that 40% of patients had molecularly undetectable *BCR-ABL1* transcripts over 6 months [134]. A phase II trial using ruxolitinib alone showed 60% ORR, where 33% observed clinical benefit in one or more categories: platelet count improvement, hemoglobin improvement, ≥50% reduction in spleen size and ≥50% reduction in symptoms [135]. A phase I/II trial in combining ruxolitinib with CML-specific TKIs showed achievement of CCyR in 87.5% and MMR in 37.5% of patients [136].

### 4.8. Targeting PPARγ/STAT5/HIF2α Axis

STAT5 activation leads to the induction of hypoxia inducible factor-2α (HIF-2α)/CITED pathway for adaptation in low oxygen levels of the BMM to maintain LSC dormancy and self-renewal potential [137,138,139,140]. PPARγ, a negative regulator of the STAT5/HIF-2α/CITED pathway inhibits adhesion of LSCs to the extracellular matrix and drives apoptosis [8,137,140,141]. Preclinical studies demonstrated that thiazolidinediones (PPARγ agonists) upregulate matrix metalloproteinase-9 (MMP-9) and MMP-2 to inhibit LSC invasion and adhesion to the BMM. They also activate caspase-3 for LSC apoptosis [8,137,140,141]. Other findings include upregulation of PPARα ligands e.g., clofibrate and enhanced expression of human organic cation transporter 1 (hOCT1) via WY-12643, which increase cellular uptake of TKIs to promote TKI-mediated apoptosis [139,141]. Preliminary clinical studies in 3 CML patients showed that combined use of pioglitazone and IM accomplished sustained complete molecular remission for up to 4.7 years in all patients, even after pioglitazone withdrawal [141]. Phase II ACTIM trial showed that IM and pioglitazone had no drug interactions, yet their combination achieved MR4.5 at 12 months in 56% of patients [138]. Novel STAT3 inhibitor BP-5087, derived from SF-1-066, demonstrated 10-fold greater potency in reducing STAT3 phosphorylation and translocation, inhibiting survival of TKI-resistant CML cells and LSCs in preclinical studies [18,142]. Combination with TKIs showed dramatic increase in effects, whereas monotherapy of either was evidently inferior [142]. However, STAT3/5 inhibition may be less effective than JAK inhibition as other STATs may compensate for STAT3/5 loss [143].

### 4.9. Prostaglandin E (PGE) 1 Analogue

PGE2 is a pro-inflammatory prostaglandin upregulated by BCR-ABL1 [17,144]. It promotes β-catenin nuclear accumulation, stabilization and localization to promote the β-catenin/Wnt signalling, conferring to LSC stemness, TKI-resistance and disease progression [17,144]. On the contrary, PGE1 exhibits protective functions against LSCs [17,144]. Preclinical studies showed that misoprostol, a PGE1 analogue acts via EP4 receptor to inhibit Tcf1/Lef1 and Fos/FosB, hence reducing LSCs by >10-fold [17,144]. Exhibiting negligible effects on normal HSCs, the activation of PGE1 poses as a promising target for CML stem cell eradication.

### 4.10. Activation of Promyelocytic Leukaemia—Nuclear Bodies (PML-NB)

Promyelocytic leukaemia (PML) forms PML-NBs to repair DNA double-strand breaks (DSBs), maintain telomere homeostasis and maintain normal HSC asymmetric division through the PML/PPAR/FAO pathway [17,145,146]. Preclinical studies showed that PML upregulation in mesenchymal stromal cells upregulated inflammatory cytokines (IL-6/IL-6R and CXCL1/CXCR2), which are crucial for the maintenance in the BMM and TKI-resistance of CML stem cells [146]. Arsenic trioxide (ATO) was used as a first-line treatment for CML before the development of TKIs, but preclinical studies showed limited effectiveness in targeting CML stem cells as a single agent [147,148,149]. However, combination with TKIs showed LSC targeting, downregulation of VEGFR and angiogenesis, upregulation of NKG2D ligands to induce NK-cell mediated cytotoxicity, growth arrest, inhibition of RAS/MAPK and PI3K/AKT pathways, and apoptosis via extrinsic pathways (caspase-8/-10, TNFR1) and intrinsic (BAX) pathways and the induction of ER stress [147,148,149]. ATO/IFNα combination therapy demonstrated superior in-vivo and in vitro results compared to ATO/TKIs, where it induced cell-cycling of dormant LSCs and inhibited the Hh pathway, hence, leading to autophagy-induced cell death [145]. The established ability of ATO/IFNα to overcome TKI-resistance and abolish CML stem cells in preclinical studies [145,150] has led to phase I clinical trials [151]. In a cohort of eight patients, decrease in *BCR-ABL1* fusion transcript was seen in 100% and 87.5% patients after trial and 12 months after trial respectively. MR4.5 or above was achieved in 87.5% and 55.6% patients immediately after study and 12 months later, respectively [151].

## 5. Targeting the CML Stem Cell Microenviroment, Survival and Self-Renewal

Normal HSCs interact with endothelial cells, neural cells, osteoclasts, mesenchymal stromal cells and osteoblasts in the BMM [17,25,152,153]. Selectins, integrins, and CD44 expressions are required for HSC engraftment and adhesion between fibronectin on the extracellular matrix and CD106 (VCAM-I) on the BM endothelium [23,25,152,153,154]. HSC rolling and homing is mediated by interaction between constitutively expressed E- and P- selectins and VLA4, where SDF1 and its receptor CXCR4 acts as a chemo-attractant through β1/2− integrins and SDF1 for stable engraftment [23,25,152,153,154,155,156]. BCR-ABL1 impairs the SDF1/CXCR4 axis in normal HSCs but upregulates it in CML stem cells, conferring selective homing and survival in the BM niche [17,23,152,153,157,158]. In addition, CML stem cells have defective β1-integrin levels (VLA4 or VLA5), allowing redistribution and mobilization into the PB and other organs, e.g., spleen with the potential of uncontrolled extramedullary myeloproliferation and local LSC reservoirs [152,154,157]. CML stem cells alter extrinsic factors and upregulate expression of CD44^+^ and E-selectin to promote prominent BMM changes such as marrow fibrosis for exclusive stem cell engraftment and dormancy, offering protection from drug-targeting [25,152,154,158,159,160,161,162].

### 5.1. Dipeptidyl-Peptidase (DPP-4) Inhibition

DPP-4 (CD26) is an overtly expressed protease on LSC surface, where it cleaves the SDF1/CXCR4 axis to facilitate LSC mobilization into the PB independent of niche regulations [16,17]. TKIs decrease CD26^+^ LSCs but levels dramatically increase following resistance or relapse [16,17,137]. CD26 is not expressed on normal HSCs, suggesting that it may be a marker for LSCs as concentrations correlate with white blood cell (WBC) count [16]. DPP-4 inhibitors (gliptins) normalize the dysregulated SDF1/CXCR4 axis to restore and promote homing of LSCs [16,36]. Interestingly, Willmann et al. demonstrated that single agent nilotinib could inhibit engraftment and induce apoptosis of LSCs, while neither vildagliptin nor imatinib addition exhibited these effects [36]. Combination of nilotinib with vildagliptin also did not produce cooperative results, suggesting insignificant effects of co-administration [36]. However, vildagliptin alone reduced disease expansion through limiting LSC mobilization [16,36]. In samples of two nilotinib-pretreated CML patients with diabetes mellitus using gliptins for diabetic control, BCR-ABL1 transcripts were near undetectable or undetectable [16].

### 5.2. E-Selectin Antagonist

Uproleselan (GMI-1271) is an E-selectin inhibitor which dislocates homed LSC from the BM niche into PB for cellular differentiation [154,163]. Promising phase III study results in acute myeloid leukaemia (AML) for LSC eradication [163] has led to preclinical studies in CML. In vitro studies demonstrated cell cycle progression via upregulated CDK6 (cell cycle promotor) and downregulated p16 (cell cycle inhibitor), leading to an increase in cells in G-phase and increase G_2_/S/M phase when used as monotherapy or in combination with IM [152,154]. Reduced CD44^+^ expression via the Scl/Tal1 pathway, increased CML stem cell cycling, and restoration of TKI-sensitivity were also noted [152,154]. Murine models showed depletion of LSC and *BCR-ABL1*^+^ cells, spleen size reduction, impaired LSC engraftment to the BM and spleen, and improved OS [152,154].

### 5.3. Targeting SDF1/CXCR4/CXCR7 Axis

Preclinical studies showed that disruption of the SDF1/CXCR4/CXCR7 axis of mesenchymal stromal cells reduced EZH2 expression [164], increased self-renewal capacity in LSCs and the ability to override TKI-resistance with no effect on osteoprogenitor cells, mesenchymal stromal cells and normal HSCs [164,165,166]. NOX-A12, a pegylated Spiegelmer, inhibits SDF1 and antagonizes the SDF1-CXCR4 or -CXCR7 interactions to inhibit LSC homing and causes TKI-sensitization [167]. In-vitro studies showed enhanced abolishment of SDF1-mediated migration in BCR-ABL1^+^ cells and induction of apoptosis when combination with imatinib was used (*p* < 0.00005) [168]. In-vivo studies showed eradication of FLT3-ITD^+^ cells and inhibition of SDF1-mediated migration of FLT3-ITD^+^ cells [168]. Plerixafor, an allosteric CXCR7 agonist and CXCR4 antagonist/partial agonist, is clinically used for stem cell mobilization in HSCT in multiple myeloma and non-Hodgkin lymphoma [165,168,169]. Its use in in vitro studies with K562 and KU812 cell lines showed reduction of drug-resistance, cellular migration and adhesion to BMM and sensitization to TKI [165]. Plerixafor in in-vivo murine models mobilized LSCs to the PB, potentiating TKI-induced tumor bulk eradication [165]. However, Agarwal et al. presented contradicting in-vivo results, which demonstrated that TKI plus plerixafor led to stem cell infiltration of the central nervous system (CNS) and subsequent development of neurological deficits [170].

### 5.4. Hypoxia-Inducible Factor (HIF) Targeting

HIFs interact with HIF-responsive elements (HRE) for gene regulation, depending on the oxygen concentration of the microenvironment [17,28,171,172,173]. The hypoxic BM niche contains high ROS levels which causes upregulation of HIF-1α and HIF-2α, suppressing BCR-ABL1 oncoprotein yet permitting CML stem cell survival, quiescence, immune-evasion, TKI-resistance and potential transformation of normal HSC into induced pluripotent stem cells capable of becoming LSCs [17,28,172,173]. This is mediated by glucose transporter 1 (GLUT1) and tumour M2-pyruvate kinase (PKM2), which lead to increased glycolytic flux [28], p21 upregulation for cellular proliferation [172], suppression of p53 [28,172,173], increased transcription of antioxidant enzymes (FoxO and Nrf2) [28,173], overt Oct4 and c-Myc for transformation of other haematopoietic cells into LSCs [173], and evasion of cellular immunity through B7H1/programmed death ligand 1 (PD-L1) expression, and soluble factors such as nitric oxide [28]. LSCs have high enough ROS levels for clonal evolution yet low enough levels to maintain stemness [8,17]. Acriflavine, a HIF-1 inhibitor prevents dimerization of the HIF complex and reduces LSC formation, maintenance, survival and stemness through three mechanisms [17,173,174]: depleting c-Myc at mRNA and protein levels, promoting expression of tumour suppressors (e.g., p57, p19^Arf^ and p16^Ink4a^) and inhibiting genes that favour LSC stemness (e.g., NANOG, Oct4, Sox9). In vitro studies using K562, KCL22, and LAMA-84 CML cell lines and in-vivo studies demonstrated cytotoxicity against BCR-ABL1^+^ cells and LSCs, where adverse effects on normal HSCs were significantly less-severe in-vivo [174].

### 5.5. Targeting Hh Pathway

Hh homologues bind to the Patched (PTCH) receptor, activating Smoothened (Smo) and Gli family of transcription factors to mediate downstream signalling (e.g., Myc, cyclin-D1, Bcl-2, SOX2) for cellular regeneration and homeostasis [8,18,29,175,176]. Low Shh levels in mesenchymal stromal cells along with hyperactivation of Shh and Smo in CD34^+^ and c-kit^+^ (*p* < 0.05) LSCs stimulate cyclin-D1-mediated LSC quiescence, maintenance and uncontrolled expansion through the Wnt/β-integrin pathway [175]. Hh overactivation is seen in 50% chronic phase (CP)-CML, 70% accelerated phase (AP)-CML and >80% blast-phase (BP)-CML patients [175]. In vitro and vivo studies showed that LDE225 (sonidegib), a highly selective and potent Smo inhibitor, was effective alone and in combination with TKIs in eradicating Hh-mediated self-renewal capacity of CD34^+^ and BCR-ABL1^+^ CML cells [18,177]. In a phase I trial CA180323, another Smo inhibitor, BMS-833923, was investigated in combination with dasatinib, showing no drug interactions and undesirable toxicity profiles and minimal reduction of *BCR-ABL1* progenitors [176]. Although preclinical and preliminary clinical studies showed conflicting results, the Hh pathway remains a significant target worth investigating.

### 5.6. Targeting Wnt/β-Catenin Signalling

Porcupine (PORCN)-dependent acetylation of Wnt ligands is essential in Wnt/β-catenin signalling for maintenance of cellular functions [17,178,179]. *BCR-ABL1* drives constitutive secretion of Wnt-ligands and overexpression of frizzled-4 (FZD4) receptors to promote nuclear transduction and stabilization of β-catenin, mediating TKI-resistance [178,179,180]. Riether et al. proposed that it might be induced by prolonged TKI exposure as TKI-therapy depleted miR29 and amplified CD70 expression, leading to CD27-mediated Wnt activation for LSC quiescence and therapy resistance [181]. In an in-vivo study using transgenic murine models with CD34^+^ and c-kit cells, potent PORCN inhibitor WNT974 in combination with nilotinib was efficacious in reducing neutrophils, white blood cells and myeloid cells in PB, with eradication of CML stem cells and other progenitors in the BM and spleen [18,178,179]. Mice treated with nilotinib monotherapy died after 30 days while nilotinib plus WNT974-treated mice had prolonged survival with prominent suppression of c-Myc, cyclin-D1 and Axin-2 expression [18,178]. C82, a β-catenin inhibitor, downregulated CD44, c-Myc, STAT5, survivin, and CRKL in T315I and E255V mutant cell lines, eliminating LSCs in-vitro and in-vivo [27].

### 5.7. Targeting Protein Phosphatase 2A (PP2A)

PP2A is a serine-threonine phosphatase that acts as a tumour suppressor, contributing to >90% intracellular phosphatase activity alongside PP1 [29,182,183,184,185]. PP2A dephosphorylates Myc, disrupting Myc/MAX interaction and inhibits gene expression for mitochondrial biogenesis. BCR-ABL1 oncoprotein amplifies endogenous expression of potent PP2A inhibitors protein SET (SET), cancerous inhibitor of PP2A (CIP2A) and PP2A-Aα that inactivate phosphatase activity, hence, resulting in high levels of Myc and uncontrolled DNA synthesis for LSCs survival and maintenance [29,182,183,186,187]. The Myc/MAX complex can directly bind BCR-ABL1 to upregulate its mRNA and protein content, establishing a positive feedback loop for LSCs [182,185]. Myc inhibitor 10058-F4 suppressed CIP2A in 80% of CD34^+^ cells (*p* = 0.04) and 85% of K562 cells (*p* = 0.01), preventing Myc/MAX interaction and restoring tumour-suppressor functions in vitro K562 and CD34^+^ cell lines [29,184]. However, Myc-targeting remains a therapeutic challenge due to the lack of a clear ligand binding domain [182]. OP449, a SET antagonist reactivated PP2A and significantly reduced JAK/STAT5 and PI3K/AKT pathways in vitro CD34^+^ and K562 CML cells, as well as alleviated tumour burden in vivo xenografted mice with human CML cells [182,186]. FTY720, a SET antagonist activates extrinsic and intrinsic apoptotic pathways in a PP2A-dependent manner [29,184,186,188] or via the activation of caspase-3/-8/-9 and pro-apoptotic BH3-only proteins (BIM and BID) in-vitro K562, MYL, KBM5 and KCL22 cell lines [184]. It can also overcome BIM-deletion-mediated, Gal-3-mediated *BCR-ABL1* kinase-domain-mediated TKI resistance, with synergistic activity in combination with imatinib [184]. However, Bcl-2 expression partially hinders FTY720-mediated apoptosis [184]. Combination of FTY720 or OP449 with TKIs showed drug synergism [182,184,186,188].

## 6. Targeting CML Stem Cells via Epigenetic, Ribosomal and Transcriptional Regulation

Epigenetic modifications are reversible and heritable changes that regulate DNA expression while maintaining the same nucleotide sequence [189,190,191]. High ROS levels and hypoxic conditions of the BMM lead to DNA damage and ineffective repair, making LSCs prime candidates to undergo genetic evolution. Pre-leukaemic lesions in epigenetic regulators (e.g., *DNMT3A*, *IDH1/2*, *TET1/2*, *TP53*) result in clonal hematopoiesis of intermediate potential (CHIP), a predisposing factor for haematological malignancies [8,192,193,194,195]. Despite having a peak incidence of 15–20% in the general healthy population after the age of 70 [8,196,197,198,199], CHIP is not a cause for CML [8,21,193]. However, the concurrent presence of CHIP and leukaemia drives LSC transformation and survival, and is associated with an inferior prognosis [8,17,189,195,196,197,198,199].

### 6.1. Bromodomain and Extra-Terminal (BET) Inhibitor

BET proteins are epigenetic regulators of transcription, inflammatory processes, and cell-cycle regulation [18,200,201,202]. Bromodomain-containing protein 4 (BRD4) is the only ubiquitously expressed member of the family directly bound to P-TEFb to maintain chromatin stability and G_2_/M phase transition in the cell cycle. BCR-ABL1 and LSCs can acquire secretory-associated senescent phenotype (SASP) to drive BRD4 activity and upregulation of Myc, leading to overt release of pro-inflammatory cytokines (e.g., IL-1, IL-6, IL-8, IL-17, IL-23, BMP2, TNFα, CCL9, NF-κB) to favour LSC senescence. Aberrant BRD4 activity also induces active transcription and expression of PD-L1 on leukaemic cells, myeloid dendritic cells and macrophages for immune-evasion. BRD4 inhibitor JQ-1, as well as BRD4 degraders dBET1 and dBET6 have shown to be promising and potent inhibitors that downregulate Myc in targeting LSCs and overcoming TKI-resistance [200,202,203,204,205]. In vitro studies showed that JQ-1 increased IL-12β to reduce VEGFR-mediated angiogenesis while decreasing PD-L1 expression to promote CTL-cytotoxicity [200,201,202,204] and IL-6-mediated Jagged1/Notch1 cellular invasion and migration [201]. In vitro studies using K562 and KU812 cell lines showed superior potency of dBET6 and dBET1 over JQ-1 in suppressing BRD4 and Myc expression, where dBET6 and dBET1 could eliminate BCR-ABL1^+^ cells and CD34^+^/CD38^−^ LSCs while JQ-1 failed [202]. In vivo studies demonstrated that dBET6 could override niche cell-induced TKI-resistance in CML LSCs, while JQ-1 was only able to restore TKI effects completely in KU812 cells and partially in K562 cells [202]. All three BRD4 inhibitors inhibited IFNγ-induced PD-L1 expression in LSCs [202,205]. Promising preclinical studies have led to the development of novel BRD4 inhibitor CPI-0610, which is currently in phase I clinical trials (ClinicalTrials.gov identifier: NCT02158858).

### 6.2. EZH2 Inhibition

EZH2 is a histone methyltransferase and a component of the polycomb repressive complex 2 (PRC2) for histone H3 methylation (H3K27me3) and transcription inactivation [17,206,207,208]. EZH2 hyperactivity blocks myeloid differentiation to promote LSC expansion, survival and TKI-resistance [17,43,207,208,209,210,211]. In vitro studies showed that EZH2 inhibitor EPZ-6438 upregulated tumour suppressor p16 to deplete leukaemic cells in K562, HEL, Kasumi-1, ME-1, Mv4-11 and MOLM13 cell lines [207,210]. Inactivation of EZH2 showed significant reduction in WBC count and LSCs in the PB, prolonged survival, and absence of splenomegaly and pulmonary hemorrhage compared to the control arm [210]. Another in vitro study showed 20–40% reduction in CD34^+^ cells and 60–80% reduction in progenitor granulocyte/erythroid/megakaryocyte/macrophage (GEMM) and total colony forming cell (CFC) [43]. Combination with TKI increased activation of H3K27me3 targets (e.g., CDKN2A) and upregulated pro-apoptotic targets of p53 (e.g., NOXA, p53 upregulated modulator of apoptosis (PUMA), BAX, CDKN2A, TNFRS10B), showing >70% reduction in CD34^+^/CD38^−^/CD45^+^ cells, near complete eradication of CD34^+^/CD45^+^ cells and restoration of TKI-sensitivity [43,211]. Murine xenograft models with CD34^+^ cells showed similar findings [43].

### 6.3. Histone Deacetylase (HDAC) Inhibitor

HDAC packs histones tightly to inhibit transcription. Aberrant activity in CML LSC halts myeloid differentiation to allow LSC survival [18,212,213,214]. Panobinostat (LBH589), a HDAC inhibitor combined with TKIs in preclinical studies showed impairment of LSC quiescence and engraftment, promoting TKI-mediated apoptosis [18,212,213,214] in vivo CD34^+^ mice [212,213] and in vitro cell lines CD34^+^/CD38^−^, K562, K562/IM-R1, Ba/F3 and Fa/F3/T315I [18,212,213]. A phase I clinical trial exhibited no dose-limiting toxicities and 44% patients achieving >1 log reduction in *BCR-ABL1*/*ABL1* transcripts, but was discontinued due to slow accrual [18]. A phase II study also showed no MCyR nor molecular response. However, in light of encouraging preclinical results, a phase Ib trial is currently underway (ClinicalTrials.gov identifier: NCT03878524). Chidamide, an orally bioavailable HDAC inhibitor in vitro increased acetylation of Histone H3, activated caspase-3/-9 and decreased levels of β-catenin, surviving and Myc in CD34^+^ cells, inducing apoptosis [215]. It also exhibited limited toxicity to normal HSCs, and produced drug-synergism in combination with TKIs in overriding BCR-ABL1-dependent and -independent mutations [215]. Another novel pan-HDAC inhibitor MAKV-8 reduced c-Myc expression, activated caspase-3/-9-mediated apoptosis and triggered ER stress for LSC eradication using K562 and MEG-01 cell lines in vitro [214]. When used in combination with TKIs, in vivo K562 cells in zebra-fish were completely abolished [214].

### 6.4. Protein Arginine Methyltransferase (PRMT5) Inhibitor

PRMT5 catalyzes histone methylation for RNA metabolism, transcriptional regulation, ribosome biogenesis and cell-cycle regulation [216]. Aberrantly expressed PRMT5 binds to BCR-ABL1 to form a positive feedback loop, which is associated with worse progression-free survival in CML patients [216,217,218]. Preclinical studies showed that PJ-68, a PRMT5 inhibitor suppressed the Wnt/β-catenin pathway and induced negative regulators of LSC cellular renewal P15^INK4B^ and p27^KIP1^ for CD34^+^CD38^−^ apoptosis [18,218].

## 7. Targeting CML Stem Cells via P53 Modulation

P53 is crucial in tumour suppression and apoptotic control [29,219,220]. It is dysfunctional in CML as it directly binds to IκBα and BCR-ABL1, leading to dysregulated p53 and Myc levels [29,219,220]. Preclinical studies showed that depletion of E3 ligase FBWX7 (SCF) led to Myc-induced p53 upregulation, promoting p53-mediated cell-cycle regulation and apoptosis in CD34^+^ and HeLa cell lines [219]. This spared normal HSCs and achieved near complete elimination of BCR-ABL1^+^ cells and LSCs [219]. Dasatinib showed some efficacy in STAT5 inhibition and mutant TP53 reduction. However, it was insufficient to eliminate LSCs [219,221]. RITA (NSC652287) binds and prevents p53 degradation. In vitro studies showed degradation of IκBα and downregulation of NF-κB-regulated proliferative (c-Myc) and anti-apoptotic (Bcl-2, XIAP, cIAP1) genes, inhibition of PI3K/AKT and JAK/STAT5 signalling pathways, increased p53-mediated apoptosis and decreased c-Myc levels leading to subsequent CD34^+^ and K562 cellular knockdown [219,222,223]. In-vivo mouse CML models using RITA in combination with CPI-203 (BET family inhibitor) showed decreased levels of CD11b, CD19, CD33, CD34 and CD45, suggesting reduced LSC engraftment [219,222]. P53-mediated apoptosis requires phosphorylation at Serine-46 (Ser46), where deficiency leads to drug resistance. In vitro studies showed that, despite the ability of RITA to override HDM2 inhibition, it remained ineffective against p53-mutant cells lacking phosphorylated Ser46 [224,225].

### 7.1. Sirtuin 1 (SIRT1) Inhibition

SIRT1, a NAD^+^-dependent deacetylase, is a potent suppressor of tumour suppressor p53 found to be overly expressed in CML stem cells [18,221,226]. It activates PGC-1α to promote mitochondrial DNA replication to maintain the bioenergetic demands of LSCs [226]. SIRT1 deletion in vitro and in vivo demonstrated downregulation of mitochondrial genes and upregulation of p53 acetylation in LSCs and progenitor cells [221,226]. While TKI treatment did not affect mitochondrial respiration [226], combination with SIRT inhibitors restored sensitivity to TKIs and subsequent TKI-mediated apoptosis [221,226].

### 7.2. Human Double Minute 2 Protein (HDM2) Inhibition

HDM2, another p53 negative regulator, inhibits TP53 transcription via binding to its transactivation domain [18,227,228]. Hyperactivity of CML LSCs leads to p53 proteasomal degradation and evasion of apoptosis. DS-5272, an HDM2 antagonist, restored TKI sensitivity via p53 reactivation and induction of NOXA, leading to silencing of anti-apoptotic Mcl-1 [229]. Combination with TKIs or BET inhibitors suppressed downstream Myc-related pathways and upregulated p53, NOXA and BAXA, reducing the threshold for TKI-mediated apoptosis [229]. In vitro and in vivo results demonstrated high selectivity and near complete eradication of LSCs [229]. MI-219 directly stabilized and reactivated p53, reduced CD44^+^ for LSC homing and engraftment, and depleted important genes for LSC self-renewal (e.g., JARID2, PRDM16) both in vitro and in vivo [18,227]. MI-219 had limited effects on normal HSCs, and it upregulated IFNAR1 to drive LSCs into the cell cycle and exhaust them [227].

## 8. Targeting Autophagy in CML Stem Cells

Autophagy is the stress-induced formation of autophagosome for recycling and degradation of damaged and/or aged cytoplasmic components to sustain bioenergetic and nutritional demands [24,29,230,231,232]. A metabolic shift in LSCs results in increased glucose influx, pyruvate shuttling, glycolysis, anaplerosis, oxidative phosphorylation, and ROS overload (Warburg effect) for survival and maintenance of stemness [8,29,171,232]. Moreover, the upregulation of Beclin-1 is essential in autophagic flux [29,230,231], acting as a protective mechanism to avoid oxidative stress and apoptosis for the maintenance of stemness [24,29,230,231,232].

### 8.1. Tigecycline

Tigecycline, a third generation tetracycline, is active against LSCs via three mechanisms with negligible effects on normal HSCs [8,203,232,233,234,235]: Atg7 knockdown depleting glucose levels in LSCs, downregulation of signalling pathways (e.g., Wnt/β-catenin, PI3K/AKT/mTORC1, p21^CIP1^/Warf1, hypoxia-inducible factors (HIF), c-Myc) for autophagosome formation, and binding to 28S subunit of ribosome (homologous to 30S in bacteria) to activate cytochrome-c/caspase-9/caspase-3 causing defective mitochondrial translation, oxidative phosphorylation, electron transport chain and oxygen consumption. In vitro studies using CD34^+^/CD38^−^ cells and in vivo murine models demonstrated superior efficacy in the reduction of tumour load in combination with IM than either agent alone [233].

### 8.2. Chloroquine (CQ)

CQ becomes protonated and trapped in lysosomes to alkalize acidic hydrolases, prevents fusion with autophagosomes, increases cellular stress and drives apoptosis [236]. In vitro studies showed that CQ eliminated BCR-ABL1^+^ cells and sensitized CD34^+^/CD38^−^ to TKI-mediated apoptosis [24,237]. These lead to CHOICES (Chloroquine and Imatinib Combination to Eliminate Stem cells), a randomized phase II trial in comparing IM alone versus IM plus hydroxychloroquine (HCQ) [24,238]. MMR was 92% (IM/HCQ) vs. 80% (IM) at 12 months, and the qPCR level at 24 months with ≥0.5 log reduction was 75% (IM/HCQ) versus 67% (IM) [238].

## 9. Immunotherapeutic Targeting of CML Stem Cell

### Targeting PD-1/PD-L1 Axis

The PD-1/PD-L1 axis is responsible for self-tolerance [44,239]. CML induces IFNγ-mediated PD-L1 expression for LSCs to aid evasion of CTL-cytotoxicity and recruitment of MDSCs and regulatory T cells for immune-evasion. Preclinical studies showed that T-cell immunotherapy with PD-1 inhibition eliminated LSCs [239]. Nivolumab, a monoclonal IgG4 antibody (Ab) against PD-1, was used on an 82-year-old man, with the ability to achieve undetectable *BCR-ABL1* transcripts as a single agent [240]. Results of phase I clinical trials with dasatinib are pending (ClinicalTrials.gov identifier: NCT02011945). Avelumab [241], a monoclonal IgG1 Ab against PD-L1, is currently in clinical trials with various TKIs (ClinicalTrials.gov identifier: NCT02767063).

## 10. Novel Therapies Targeting MPN Stem Cells via Signaling, Apoptotic and Cell Cycle Pathways

### 10.1. Telomerase Inhibition

Telomerase is a ribonuclear protein complex comprised of human telomerase reverse transcriptase (hTERT), an RNA template (hTR), and specialized proteins (e.g., dyskerin), that extend the length of telomere [86,242,243,244,245]. It maintains replicative potential and is actively expressed in HSPC [86,242,243,244,245]. In MPN, telomerase is overexpressed and upregulated [86,242,243].

Imetelstat (GRN163L) is a 13-mer oligonucleotide that inhibits hTR in telomerase [86,242,243,244,245,246], resulting in selective apoptosis of MPN stem cells [242,243,245]. Preclinical studies demonstrated preferential apoptosis of MF CD34^+^ cells irrespective of driver mutations [243], and reduced polyploidization and maturation of CD41^+^/CD42b^+^ megakaryocytes [242]. Decreased malignant megakaryocytes led to reduced growth factors (e.g., platelet-derived growth factor (PDGF), fibroblast growth factor 2 (FGF-2)) and inflammatory cytokines, hence reduction in BM fibrosis in MPN cultures [242] A pilot study suggested that additional spliceosome mutations (e.g., *U2AF1*, *SF3B1*) might lead to suboptimal telomerase upregulation and increase patients’ sensitivity to telomerase inhibition [244] (Table 3).

### 10.2. BET Inhibition

BET proteins are chromatin-reader proteins [262]. The hydrophobic N-terminal bromodomain (BRD) of BET binds to acetylated lysine on histone tails and recruits chromatin factors to regulate transcription of c-Myc, Bcl-2, and NF-κB [86,262,263,264]. NF-κB is crucial in mediating chronic inflammation in MPN [86]. BET inhibitors displace BET proteins to attenuate NF-κB signalling [262]. In-vivo studies showed BET inhibitor JQ-1 diminished NF-κB activation and pro-inflammatory cytokine production [265]. In MPN, both JAK/STAT activation and BET activation promote NF-κB pathway. Interplay between NF-κB-activated cytokines and JAK/STAT3 further contributes to disease progression [264,265]. Preclinical studies showed that combination of JQ-1 with ruxolitinib produced synergistic effects in reducing fibrosis, extramedullary haematopoiesis (EMH), leukocytosis, and targeting of primary CD34^+^ progenitor cells in MF patients [86,264,265]. In MF, 10% patients display loss of EZH2, which promotes acetylation of histone H3 at lysine 27 (H3K27ac). This increases the sensitivity of BET inhibitor JQ-1 to JAK2V617F/EZH2^Δ/Δ^ cells [264,266] (Table 3).

### 10.3. Mouse Double Minute 2 Homolog (MDM2) Inhibition

MDM2, coupled with MDM4, ubiquitylates and degrades p53 to maintain p53, a tumour suppressor gene that plays a critical role in apoptosis and DNA repair [247,267,268,269], to maintain it at a low level [247]. In MPN, MDM2 is overexpressed in PV and MF CD34^+^ cells [247,267,268,269]. Gain of 1q and 12q chromosomal rearrangements are associated with increased expression of MDM4 and MDM2 respectively [268,269]. Gain of 1q is seen in 43% MPN patents with leukaemic transformation. The presence of +1q contributes to MPN disease progression and is also found to be specifically associated with JAK2V617F mutation, while 12q gain is associated with MF [269]. MDM2 inhibitors, nutlins (Nutlin-3, RG7112, RG7388 (idasanutlin), HDM201, and KRT232), act by hindering MDM2-p53 interactions, increasing p53 levels, resulting in MPN CD34^+^ cell apoptosis [268]. IFNα can also act through p53 to mediate PV haematopoietic progenitor cell (HPC) apoptosis by activating the p38 MAPK pathway [63,247,267]. Preclinical study showed that low dose RG7112 and Peg-IFNα-2a increased p53 downstream pro-apoptotic proteins (e.g., PUMA, BAX), leading to MPN CD34^+^ cell apoptosis. The elimination of MPN stem cells was demonstrated by the significant reduction of *JAK2*V617F allele burden in PV, MF CD34^+^ cells in BM as shown in the study [267]. Clinical trials of combination therapy with ruxolitinib in PV and MF are also underway, but results are yet to be published [270,271,272].

### 10.4. Heat Shock Protein (HSP) Inhibition

HSP is a family of ATP-dependent, cytoprotective, stress-response protein chaperones that bind and stabilize client proteins in their functional active form, thus maintaining survival advantage of MPN cells. [248,272,273,274,275]. HSP90 chaperones JAK2 while HSP27 modulates STAT5 phosphorylation, making them therapeutic targets in MPN [273]. HSP90 inhibitor disrupts association between HSP90 and JAK2, leading to JAK2 misfolding and degradation by 26S proteasome [272,275]. In vitro and in vivo studies have demonstrated that HSP90 inhibitor (PU-H71) inhibited, and even degraded JAK2, resulting in abrogation of downstream signaling pathway (e.g., STAT3, STAT5, MAPK). A reduction of MPLW515L allele burden, EMH, and normalization of blood counts were seen in PU-H71-treated murine models [276]. However, preclinical study using another HSP90 inhibitor AUY922 showed a rapid elevation of *JAK2*V617F level upon AUY922 termination [275]. A phase 2 study also showed that AUY922 failed to demonstrate consistent reduction of *JAK2*V617F mutant allele burden. This suggests that HSP90-mediated JAK/STAT pathway inhibition may be short-lived [248] (Table 3). An important finding is that the combination of JAK inhibitor (TG101209) with AUY922 acted synergistically to induce apoptosis in primary MF CD34^+^ cells [273,274,275]. The pro-apoptotic effect was also seen in JAK1/2 inhibitor-resistant cells [275], suggesting a possible solution to overcome ruxolitinib resistance. An emerging role of HSP27 inhibitor (KNK437) is also displayed by its synergistic action with ruxolitinib in *JAK2*V617F cell lines and PV patient cells. In murine models, HSP27 inhibitor (OGX-427, Apartosen) reduced splenomegaly, BM fibrosis and normalized counts, reflecting the therapeutic potential of HSP27 inhibitors [273].

### 10.5. Poly-ADP-Ribose Polymerase (PARP) Inhibition

PARP is a protein involved in DNA repair. It maintains MPN cell survival alongside two other DNA repair mechanisms: BRCA1/2-mediated homologous recombination repair (HRR) and DNA-dependent protein kinase, catalytic subunit-mediated non-homologous end-joining (D-NHEJ) [277]. ROS levels are found to be elevated in MPN LSCs, predisposing cells to toxic DNA DSBs [277,278], which activate DNA repair by PARP via the recruitment of repair proteins [279]. The significance of PARP inhibitors is revealed when used in combination with ruxolitinib [277]. Ruxolitinib downregulates important molecules in HRR and D-NHEJ in cell lines of all three driver mutations and sensitizes both proliferating and quiescent MPN stem cells to PARP inhibitors. Preclinical studies demonstrated promising synergistic effects of PARP inhibitors (Olaparib and BMN673) with ruxolitinib in eradicating MPN stem cells by apoptosis [277].

### 10.6. CD123 Targeting

Interleukin-3 receptor (IL-3R) consists of an alpha chain (CD123) and a common beta chain (CD131) [86,280]. IL-3, a cytokine released by activated T-lymphocytes, binds to CD123 which dimerizes with CD131 to trigger downstream JAK2 signalling pathway [280,281]. CD123 is overexpressed in CD34^+^/CD38^−^ LSCs in AML, but not in normal HSCs [282]. Overexpression of CD123 is also found in some MF patients, especially in patients with monocytosis, which confers a poor prognosis [249,281]. Tagraxofusp is a recombinant protein genetically engineered from the fusion of IL-3 to the catalytic and translocation domains of diphtheria toxin [86,281]. Upon binding to CD123 and internalization into MPN LSCs, the catalytic domain of diphtheria toxin is cleaved. This inactivates elongation factor 2 (EF2), which is responsible for protein synthesis. Thus, the apoptosis of LSC is driven [280]. Early phase clinical trials performed to evaluate the efficacy of tagraxofusp in advanced MF patients showed promising results [249,283] (Table 3).

### 10.7. Proviral Integration Site for Moloney Murine Leukemia Virus (PIM) Kinase Inhibition

PIM kinase is a family of anti-apoptotic serine/threonine proto-oncogenes that are transcriptionally activated by JAK/STAT signalling [272,284,285,286]. In MPN, continuous activation of JAK/STAT pathway is observed, making PIM kinase a potential therapeutic target [272,286]. Although the efficacy of PIM kinase inhibitor monotherapy is limited [285], it acts synergistically with ruxolitinib to suppress JAK2 signalling effectively [285,286]. In MPN, pro-apoptotic BAD protein is phosphorylated and inhibited by PIM kinase, prolonging MPN cell survival. Therefore, PIM kinase inhibitor (AZD1208) liberates BAD protein to induce *JAK2*V617F cellular apoptosis with ruxolitinib [285]. Enhanced cleavage of DNA repair enzyme PARP was also demonstrated to promote apoptosis in a preclinical study [286]. Importantly, preclinical studies showed that PIM kinase inhibitor resensitized ruxolitinib-resistant cell lines to apoptosis [285], while combination treatment prevented disease progression of myeloproliferation and splenomegaly in murine models [286].

### 10.8. PI3K/AKT/mTOR Inhibition

The PI3K/AKT/mTOR cascade is a key downstream pathway of JAK/STAT pathway for cellular survival and proliferation [86,272,287,288]. One of the limitations of standard therapy ruxolitinib is the incomplete suppression of STAT5 phosphorylation in JAK2V617F cell lines [287]. Hence, the synergistic effect of PI3K/mTOR inhibitor and ruxolitinib is postulated. Ruxolitinib inhibits phosphorylation of STAT5a while PI3K/mTOR inhibitor dephosphorylates STAT5b via CIP2A/PP2A axis [287]. Fiskus et al. showed that synergism of dual PI3K/mTOR inhibitor (BEZ235) and JAK2 inhibitor induced preferential apoptosis in MF JAK2V617F CD34^+^ cells, while sparing normal HSCs [288]. Hence, clinical trials are being carried out [261,289].

### 10.9. Bcl-xL Inhibition

Bcl-2 family includes anti-apoptotic proteins (e.g., Bcl-xL, Bcl-2) and pro-apoptotic proteins (e.g., BAX, Bcl-2 homologous antagonist killer (BAK)). Bcl-xL heterodimerizes with BAX and BAK to exert anti-apoptotic effect [290]. In MPN, Bcl-xL is overexpressed with highest level displayed in MF, followed by PV then ET regardless of JAK2V617F mutation status [290]. Significance of Bcl-xL inhibitor is seen in the cotreatment with ruxolitinib. In-vitro studies showed ABT-737, a BH3 mimetic inhibitor that inhibits both Bcl-xL and Bcl2, acted synergistically with ruxolitinib in driving cellular apoptosis [290]. It is also important to note that Bcl-xL inhibitor is a possible treatment to overcome ruxolitinib resistance. The activation of RAS and its downstream pathways inhibit pro-apoptotic Bcl-2-antagonist of cell death (BAD). Hence, BAD cannot bind and inhibit Bcl-xL, contributing to JAK2 inhibitor resistance [291]. Resensitization in JAK2 inhibitor-resistant cells was manifested by co-treatment of JAK2 inhibitor and ABT-737 in preclinical study [292]. Phase 2 clinical study has demonstrated promising results with combination treatment [293].

## 11. Targeting MPN Stem Cells via Epigenetic Regulation

### 11.1. Lysine Specific Demethylase-1 (LSD-1) Inhibition

LSD-1 is an epigenetic enzyme which demethylates lysine residue on histone H3 to sustain MPN LSC self-renewal [272,294]. In MPN, LSD1 is overexpressed and accounts for 58% of MF patients [272]. An LSD-1 inhibitor, bomedemstat (IMG-7289), is shown to markedly decrease mutant allele burden by TP53 activation and cell cycle arrest [294]. IMG-7289 restores *TP53* methylation, leading to an increase in PUMA to induce apoptosis. Meanwhile, anti-apoptotic Bcl-xL is suppressed by TP53, further facilitating apoptosis and reducing mutant allele burden in murine models. Other promising effects include the reduction of BM fibrosis, EMH-mediated splenomegaly and inflammation, as well as the normalization of blood counts. Furthermore, ruxolitinib plus IMG-7289 were shown to eliminate MPN stem cells in murine models, encouraging further investigations [294]. Clinical trials of IMG-7289 are currently underway (Table 3).

### 11.2. PRMT5 Inhibition

PRMT5 functions by methylating histones and cellular proteins such as p53 [295]. In MPN, particularly PV, PRMT5 is phosphorylated by JAK2V617F [295,296]. The phosphorylation activity inhibits PRMT5 methylation activity, and promotes clonal myeloproliferation [295]. PRMT5 inhibitor binds and impairs phosphorylation of PRMT5 by JAK2V617F, impairs E2F transcription factor 1 (E2F1) methylation, and results in downstream epigenetic dysregulation [295,296]. In addition, a preclinical study demonstrated that PRMT5 inhibitor (CTx034) abrogated erythropoiesis in JAK2V617F cells without suppressing normal haematopoiesis [297]. Erythroid progenitors were significantly reduced by another PRMT5 inhibitor (C220) [296]. Decrease in hepatosplenomegaly, marrow fibrosis, and proinflammatory cytokines were also displayed in JAK2V617F and MPLW515L murine models. These effects were further enhanced by the combination with JAK inhibitor [296]. Phase 1 clinical trial is currently underway [255] (Table 3).

### 11.3. HDAC Inhibition

HDAC is a family of proteins that diminish acetylation of histones and non-histone proteins (e.g., p53, NF-κB, HSP90, STAT3, Ku70 (enzyme for DNA repair)) [272,298]. In MPN, particularly MF, HDAC is overexpressed, inducing a number of downstream pathways [298], leading to myeloproliferation. Similar to HSP90 inhibitors, HDAC inhibitors (givinostat and panobinostat) acetylate HSP90, prevent JAK2V617F-HSP90 interactions, and degrade JAK2V617F [74,298]. A preclinical study showed that givinostat acetylated and downregulated transcriptional factor nuclear factor, erythroid 2 (NFE2), hence, reducing erythroid differentiation in CD34^+^ cells [299]. The aforementioned pathway is independent of JAK2/STAT5, which is targeted by JAK2 inhibitors [299]. Panobinostat was shown to act synergistically with JAK2 inhibitor (TG101209) to drive apoptosis in primary CD34^+^ MF cells [300]. Efficacy is further demonstrated in a phase Ib clinical trial [301]. Although HDAC inhibitors exert multiple effects, poor tolerability remains a major issue [254,272] with the exception of treatment with givinostat in PV patients, which showed positive results in a few clinical trials [74,253,284] (Table 3).

## 12. Targeting the MPN Stem Cell Niche and Marrow Microenvironment

The BM niche is essential for sustaining self-renewal of MPN stem cells, hence, providing them with survival advantages over normal HSCs via generation of ROS pro-inflammatory cytokines [14,302]. In view of the positive feedback loop between BM niche and MPN stem cells, various novel agents are developed.

### 12.1. β-3 Sympathomimetic Agonists

Nestin^+^ mesenchymal stem cell (MSC) is one of the three major types of perivascular stromal cells that secrete high levels of SDF1 for HSC quiescence and migration [302,303]. This SDF1 release negatively regulates the proliferation of JAK2V617F HSCs [303], and its expression is inhibited by the action of noradrenaline, which is released by sympathetic nerve fibres, on the β-adrenergic receptors of nestin^+^ MSCs [304,305]. In MPN, LSCs secretes IL-1β, leading to Schwann cell death and subsequent sympathetic neuropathy [256,303,305]. This results in apoptosis of nestin^+^ MSCs [256]. β-3 sympathomimetic agonist restores sympathetic regulation of nestin^+^ MSCs. In JAK2V617F murine models, β-3 sympathomimetic agonist (BRL37344) rescued nestin^+^ MSC, reduced BM fibrosis and diminished MPN HPCs [256,303,305]. Mirabegron, another β-3 sympathomimetic agonist, showed some encouraging results in a phase II clinical trial [256] (Table 3).

### 12.2. Targeting Transforming Growth Factor-β (TGF-β)

BM fibrosis is a notable feature in MPN which involves increased matrix synthesis and decreased matrix degradation [306]. In MPN, TGF-β, coupled with FGF and PDGF, are secreted by neoplastic megakaryocytes. This results in collagen production and tenascin, fibronectin and proteoglycan deposition, increasing matrix synthesis [306,307]. TGF-β also downregulates matrix metalloproteinase (MMP) and upregulates tissue inhibitors of metalloproteinase (TIMP) to inhibit matrix degradation [306]. TGF-β receptor I kinase (ALK5) inhibitors, galunisertib, and SB431542 antagonize TGF-β via ALK5/mothers against decapentaplegic homolog 3 (Smad3) pathway [308,309]. Preclinical studies have shown reduced collagen deposition, BM fibrosis and splenomegaly upon galunisertib treatment [308]. Activin receptor IIA ligand trap (ActRIIA) (e.g., luspatercept, sotatercept) is another novel agent that sequester TGF-β superfamily ligands (e.g., GDF11, Activin A) to restore terminal erythroid differentiation and improve anemia. Clinical trials are reported [257,258] (Table 3).

### 12.3. Aurora Kinase A (AURKA) Inhibition

Malignant, atypical megakaryocytes in MPN suppress the expression of GATA binding protein 1 (GATA1), which is responsible for megakaryocyte differentiation and maturation [259,260,310]. MLN8237, an AURKA inhibitor, showed efficacy in enhancing GATA1 expression, promoting megakaryocyte differentiation and polyploidization, as well as reducing BM fibrosis in animal models [310]. Additionally, MLN8237 and ruxolitinib produced synergistic effects to eliminate BM fibrosis and reduced burden of immature megakaryocytes [310]. Clinical investigations of alisertib, another AURKA inhibitor, demonstrated promising results [259,260] (Table 3).

### 12.4. Antifibrotic Therapy

The pentraxin family consists of C-reactive protein (CRP/PTX1), pentraxin-2 (serum amyloid P, SAP), and pentraxin-3. SAP is a 125-kD protein synthesized by hepatocytes to inhibit differentiation from monocytes to fibrocytes and further fibrocyte proliferation [311]. Besides the release of growth factors from atypical megakaryocytes, neoplastic fibrocytes also play a vital role in inducing BM fibrosis. Analysis via quantitative allele-specific PCR showed the presence of *JAK2*V617F and *CALR* mutations in fibrocytes, but not MSCs, suggesting that these fibrocytes were derived from a malignant clone [312]. PRM-151 is a recombinant SAP which inhibits PMF fibrocyte differentiation in BM and spleen to slow down development of fibrosis [312]. Due to the unique activity of PRM-151, it is being tested in clinical trials in combination with ruxolitinib [313,314].

## 13. Immunotherapeutic Targetting of MPN Stem Cells

### 13.1. Targeting PD-1/PD-L1 Pathway

An elevated expression of PD-1/PD-L1 has been observed in all 3 types of classical Ph-negative MPNs [273,315,316]. In MPN, JAK2V617F increases phosphorylation of STAT3 and STAT5 to promote PD-L1 expression mainly on the surfaces of monocytes, MDSCs, megakaryocytes and platelets [317]. In preclinical studies, a positive feedback was shown in MDSCs, in which it interacted with T cells and resulted in IL-10 secretion by activated T cells, hence resulting in the phosphorylation of STAT3 and induction of PD-L1 expression in MDSCs [273,315,318]. Furthermore, PV and ET patients have increased toll-like receptor 2 (TLR2) levels. This activates the MEK/ERK and STAT pathway, enhancing expression of PD-L1 [273,315]. All these contribute to the oncogene-mediated immune escape via JAK2/STAT3/STAT5/PD-L1 axis. The enhanced PD-L1 expressed in MPN binds to PD-1 on T cells to suppress their cysteine metabolism. This led to anergy, decreased cell cycle activity and exhaustion of T cells [317]. Several anti-PD-1 and anti-PD-L1 antibodies are developed with ongoing clinical trials. Their results, however, were not very encouraging [319,320,321] (Table 3).

### 13.2. Peptide Vaccination in CALR Exon 9

Mutant *CALR* possesses a new and large C-terminal peptide sequence which is completely distinct from wild-type *CALR*. This warrants interests in using *CALR* as an immunotherapeutic target [322,323]. In MPN, mutant CALR is overexpressed. This reduces MHC-I assembly and loading on cell surface, and impairs CD8^+^ T-lymphocyte targeting [323]. Interestingly, it was observed that some healthy donors harboured CD4^+^ memory T-cells towards CALR epitopes [323]. Based on the above observation, a *CALR*-mutated CD4^+^ T-lymphocyte clone has been designed to induce cytotoxic effects against *CALR*-mutated cells [323]. A phase I clinical trial is underway, but no result has been released yet [324] (Table 3).

## 14. Other Potential Therapeutic Strategies Targeting MPN Stem Cells

Several new approaches have been observed to potentially target MPN stem cells, either as single agents or in combination with Peg-IFNα-2a or ruxolitinib (Table 4 and Table 5).

## 15. Conclusions

Understanding the biology of CML and Ph-negative MPNs is an important area of scientific research. With considerable data providing insights into the biology and therapeutic targeting abnormal HSCs in CML, efforts towards identifying and quantifying abnormal HSCs in CML may facilitate efforts in achieving treatment-free remissions. In Ph-negative MPNs, this understanding may shift the treatment paradigm from cytoreduction and symptom control to effective disease modification.

## Figures and Tables

**Table 1:** Tyrosine kinase inhibitors and their effects on CML stem cells.

TKI	Observations	References
Imatinib	Reduction in CD26, a specific CML stem cell marker, in circulation after imatinib treatment In-vitro enhanced mitochondrial oxidative phosphorylation upon imatinib discontinuation, leading to CML stem cell proliferation	[16,34]
Dasatinib	Lower CD34^+^ cell proliferation in dasatinib-treated cells compared to imatinib-treated group upon TKI discontinuation	[34]
Nilotinib	Induction of apoptosis in CML CD34^+^/CD38^−^ stem cells Inhibition of CML stem cell engraftment in murine models More rapid and deeper CD34^+^/lin^−^Ph^+^ cell clearance may might increase treatment-free remission	[36,37]
Bosutinib	Higher potency in inhibiting Src phosphorylation, hence inhibiting CML primitive progenitor cells compared to imatinib Growth inhibition of CD34^+^/CD38^−^ CML stem cells in combination with gemtuzumab ozogamicin	[31,38]
Ponatinib	Higher efficacy in eradicating CML-associated LSK cells than imatinib and dasatinib in murine models	[22]

TKI: Tyrosine kinase inhibitor; CML: Chronic myeloid leukaemia: LSK cells: lin−Sca-1^+^c-Kit^+^ cells.

**Table 2:** Targeting of *JAK2*V617F and *CALR*-mutated MPN by IFNα preparations.

IFNα Preparation	MPN Subtype	Observations	References
IFNα	PV, ET, MF	Preferential targeting *JAK2*V617F HSPC, especially in CD90^+^/CD34^+^/CD38^−^ HSC-enriched progenitors, compared to mature blood cells (*p* < 0.05). Faster response in homozygous *JAK2*V617F clones than that of heterozygous clones. Faster response in targeting *JAK2*V617F HSPC than *CALR*-mutated HSPC.	[71]
	*CALR*-mutated ET, MF	Sixty-two percent showed decrease in *CALR*-mutant allele burden (median decline: 29% from baseline). Nineteen percent (4 out of 21) showed MR with >50% mutant allele burden reduction.	[62,72]
Peg-IFNα-2a	*CALR*-mutated ET	Reduction of median *CALR*-mutant allele burden from 41% to 26%. Seven percent (2 out of 31) showed complete MR. Variation in responses in patients with additional molecular mutations	[73]
Ropeginterferon α-2b	PV	Preferential inhibition and reduction of *JAK2*V617F-mutated primary hematopoietic progenitors with sparing of JAK2-wild type cells.	[69]
		2. Increase in proportion of *wild*-*type* to *JAK2*V617F-mutated colonies after 12 months of treatment compared with hydroxyurea, reflecting reduction of malignant progenitors in the BM.	

PV: polycythemia vera; ET: essential thrombocythemia; MF: myelofibrosis; HSPC: hematopoietic stem and progenitor cells; HSC: hematopoietic stem cell; MR: molecular response; Peg-IFNα-2a: pegylated interferon-alpha-2a; IFNα: interferon-alpha; BM: bone marrow.

**Table 3:** Clinical trials of novel therapies as single agents for targeting Ph-negative MPNs.

Novel Therapy	MPN Subtype	Observations	References
Telomerase inhibitor (imetelstat)	DIPSS-plus Int-2/high risk MF	CR and PR: 21% (7 out of 33) (Median duration of CR: 18 months; median duration of PR: 10 months) Reversal of marrow fibrosis in all 4 CR patients. Three of them demonstrated MR. Thirty-eight percent *SF3B1*/*U2AF1* patients showed complete response, which was higher than that of patients who did not harbour SF3B1/U2AF1 mutations (4%) (*p* = 0.04)	[244]
	DIPSS Int-2/high risk MF	≥35% SVR at week 24: 10.2% in 9.4mg/kg arm; 0% in 4.7mg/kg arm Median OS: NR in 9.4mg/kg arm; 19.9 months in 4.7mg/kg arm No significant difference of OS in all 3 driver mutations (*JAK2*V617F, *CALR*, *MPL*) Higher median OS in triple-negative patients in 9.4mg/kg arm	[245]
	ET	HR: 100% MR in 88% JAK2V617F patients Reduction by 15–66% in CALR and MPL mutant allele burden	[246]
MDM2 inhibitor (idasanutlin)	PV/ET (only 1 ET patient was enrolled)	ORR after 6 cycles: 58% in idasanutlin monotherapy; 50% when combined with Peg-IFNα-2a (Median duration of response: 16.8 months) Median reduction of *JAK2*V617F VAF: 43%	[247]
HSP90 inhibitor (AUY922)	PV, ET, MF (IPSS score: ≥2)	Reduction in splenomegaly in all patients Effect to *JAK2*V617F mutant allele burden: modest	[248]
CD123 (tagraxofusp)	DIPSS-plus int-1, int-2, high risk MF	Spleen size reduction: 53% Four out of five patients who had baseline splenomegaly and monocytosis showed spleen size reduction ≥50% TSS score reduction: 45% Effect of mutant allele burden: NR	[249]
LSD-1 inhibitor (IMG-7289)	IPSS Int-2, high risk MF	SVR after cycle 1: 50% ≥50% TSS score reduction after cycle 1: 21% Reduced BM fibrosis at day 84: 15.4%	[250,251]
	ET	Ongoing	[252]
HDAC inhibitor (givinostat)	PV	ORR: 80.6% ≥35% SVR: 19.4% Reduction of JAK2V617F mutant allele burden: moderate Complete response after a median of 4 years: 11% Partial response: 89% Normalization of spleen size: 56% Reduction of *JAK2*V617F mutant allele burden: 22%	[74,253]
HDAC inhibitor (panobinostat)	PMF, SMF	Poorly tolerated. Only 45.7% complete ≥2 cycles of treatment Reduction *of JAK2*V617F mutant allele burden: 36.82% (pre-treated) to 0.86% (cycle 4) Downregulation of pro-inflammatory cytokines	[254]
PRMT5 inhibitor (PRT-543)	R/R MF	Ongoing	[255]
β-3 sympathomimetic (mirabegron)	39 JAK2 V617F MPN patients (7 ET, 21 PV, 5 PMF, 3 post-PV MF, 3 post-ET MF)	≥50% reduction in *JAK2*V617F mutant allele burden at week 24 (primary endpoint): 0%; Twenty-five percent reduction of JAK2V617F mutant allele burden is seen in 1 patient Increased levels of nestin^+^ MSCs Reduction of reticulin fibre content from grade 1 to 0.5 at week 24; the effect is restricted in patients not treated with hydroxyurea. The mechanism is unknown	[256]
ActRIIA (luspatercept)	DIPSS int-1, int-2, high risk MF	Improvement in MF-associated anemia as shown by RBC-TI and Hb increase	[257]
ActRIIA (sotatercept)	DIPSS int-1, int-2, high risk MF	Response rate: 29%	[258]
AURKA inhibitor (alisertib)	DIPSS int-1, int-2, high risk MF	Reduction of splenomegaly: 29% Decrease in symptom burden: 32% Inhibition of marrow fibrosis Normalization of megakaryocytes	[259,260]
Anti-PD-1 (nivolumab)	PMF, post-PV, ET MF, hepatomegaly, splenomegaly	Primary outcome: efficacy and clinical activity in MF Terminated due to serious adverse effects in 75% patients	[261]

DIPSS: Dynamic International Prognostic Scoring System; IPSS: International Prognostic Scoring System; PV: polycythaemia vera; ET: essential thrombocythaemia; MF: myelofibrosis; PMF: primary myelofibrosis; SMF: secondary myelofibrosis; Int-2: Intermediate-2; CR: complete remission; PR: partial remission; MR: molecular response; HR: haematological response; SVR: spleen volume reduction; NR: not reached; ORR: overall response rate; DLT: dose-limiting toxicity; VAF: variant allele frequency; TSS: Total Symptom Score; MDM2: Mouse double minute 2 homolog; Peg-IFNα-2a: pegylated interferon-alpha-2a; HSP: heat shock protein; LSD-1: lysine specific demethylase-1; HDAC: histone deacetylase; PRMT5: protein arginine methyltransferase 5; R/R: relapsed/refractory; MTD: maximally tolerated dose; MSC: mesenchymal stem cell; ActRIIA: Activin receptor IIA ligand trap; RBC-TI: red blood cell transfusion independence; TRAE: treatment-related adverse effects; AURKA: aurora kinase A. Refer to Supplementary Materials File S1 on the prognostic models of MF.

**Table 4:** Novel therapies in combination with standard treatment (interferon-alpha/ruxolitinb) in targeting Ph-negative MPNs.

Combination	MPN Subtype	Study	Observations	References
**Combination with IFNα**				
MDM2 inhibitor (RG-7112) ± Peg-IFNα-2a	PV, PMF	Preclinical	PV: Low dose (200 nM) of RG7112 and 200ng/mL Peg-IFNα-2a reduced JAK2V617F heterozygous colonies and increased *JAK2* WT colonies PV and PMF: RG7112 and Peg-IFNα-2a activated p53 pathway, resulting MPN CD34^+^ cell apoptosis PV and PMF: RG7112 and Peg-IFNα-2a reduced repopulation of MPN CD34^+^ cells in BM and spleens of NSG mice Reduction of *JAK2*V617F mutant allele burden: PV: 75%; PMF: 80%	[267]
IFNα	PV, MF	Phase II	PV: 31% remission (CR: 9%; PR: 22%) MF: 44% remission (CR: 28%; PR: 17%) Median *JAK2*V617F allele burden reduced from 47% from baseline to 12% after 2 years (CMR: 2%; PMR: 39%)	[325]
**Combinations with Ruxolitinib**				
BET inhibitor (CPI-0610) ± ruxolitinib	MF	Phase II	SVR: 94% TSS improvement: 93% ≥50% TSS improvement: 39% ≥1 grade marrow fibrosis and/or reticulin improvement: 58% Increase in Hb by 1.5 mg/dL post-baseline (CPI-0610 monotherapy: 50%; CPI-0610 + ruxolitinib: 16%)	[326]
CD123 (tagraxofusp) ± ruxolitinib	MF-AP, BP	Preclinical	Combination of tagraxofusp and ruxolitinib had a lower IC50 JAK2V617F mutant cell line compared to tagraxofusp monotherapy Reduction of colony formation regardless of HMR mutation (*TP53, ASXL1*) in both single agent and combination	[283]
Bcl-2 family protein inhibitor (navitoclax) + ruxolitinib	PMF, SMF	Phase II	>5% reduction in mutant allele burden: 42% ≥35% SVR at week 24: 29% ≥1 grade improvement in BM fibrosis: 25%	[293]
	MF	Phase III	Ongoing	[327]
	R/R MF	Phase III	Ongoing	[328]
HDAC inhibitor (panobinostat) + ruxolitinib	IPSS int-1, int-2, high risk PMF, SMF	Phase Ib	≥35% SVR at week 24: 57% ≥35% SVR at week 48: 39% ≥20% reduction in mutant allele burden at week 48: 29%	[301]
PI3K-delta inhibitor (umbralisib; TGR-1202) + ruxolitinib	MF	Phase I	Improvement in haematological parameters: 77.8% (7 out of 9) Median reduction of TSS: 33%	[289]
Pan-PI3K inhibitor (buparlisib; BKM120) + ruxolitinib	IPSS int, high risk	Phase Ib	≥ 35% SVR: 55.6% (5 out of 9) in JAK naive arm; 42.9% (3 out of 7) in prior JAK inhibitor arm	[261]
SAP (PRM-151) ± ruxolitinib	DIPSS int-1, int-2 PMF, SMF	Phase II	BM morphological response at week 72: 54% ≥25% reduction of palpable spleen size: 50% 100% reduction of TSS between week 24 to 72: 38%	[314]
	PMF, SMF	Phase II	Median reduction of spleen size at week 24: 26.1%	[313]

CR: complete remission; PR: partial remission; CMR: complete molecular remission; PMR: partial molecular remission; Mouse double minute 2 homolog; Peg-IFNα-2a: pegylated interferon-alpha-2a; IFNα: interferon-alpha; BM: bone marrow; BET: Bromodomain and extra-terminal; SVR: spleen volume reduction; TSS: Total Symptom Score; Hb: haemoglobin; AP: accelerated phase; BP: blast phase; IC50: half maximal inhibitory concentration; HMR: high molecular risks; BM: bone marrow; R/R: relapsed/refractory; HDAC: histone deacetylase; PI3K-delta: Phosphatidylinositol 3-kinase-delta; Int: intermediate; SAP: serum amyloid P.

**Table 5:** Other potential therapeutic targets in MPN in preclinical phase.

Novel Therapy	Combination	Mechanisms	Observations	References
Arsenic trioxide	Peg-IFNα-2a	In MPN, expression of *JAK2*V617F increases PML expression ATO and IFNα enhanced the formation of PML-NB, amplifying activation of p53 and the eradication of JAK2V617F MPN stem cells	Combination treatment significantly reduced JAK2V617F-mutated erythroid colonies and BFU-E progenitors, while sparing JAK2-WT progenitors ATO enhanced clearance of *JAK2*V617F-mutated LSCs by IFNα, leading to long-term disease clearance in vivo Combination treatment significantly increased PML-NB formation Marked reduction in *JAK2*V617F mutant allele burden in granulocytes, platelets and erythrocytes in vivo	[329,330]
Metformin	N/A	Activation of AMPK pathway suppresses mTOR and reduces synthesis of anti-apoptotic Mcl-1	Apoptosis of *JAK2*V617F cell lines	[331,332]
		Attenuation of JAK2V617F downstream signaling by activating B56α subunit of PP2A	Reduced JAK2 and STAT5 phosphorylation in MPN cells	[331]
	Ruxolitinib	Delayed transition from G_1_ to S phase by downregulation of cyclin D1 and upregulation of p27	Combination treatment showed significant downregulation of cyclin D1 and upregulation of p27 in PCR and qPCR	[332]
	N/A	FIBROMET (phase II clinical trial): metformin is safe and well-tolerated. Reduction in BM fibrosis was seen but the results were not statistically significant due to small sample size		[333]
XPO1 inhibitor/SINE compounds	N/A	XPO1 exports tumour suppressor proteins (e.g., p53, NPM, p27) from the nucleus to cytosol XPO1 inhibitor (KPT-330, KPT-8602) decreases NCT	Nuclear retention of p53 in MF CD34^+^ cells, leading to apoptosis and inhibition of colony formation in MF CD34^+^ cells	[334,335]
	Ruxolitinib		Co-treatment showed greater diminution of *JAK2*V617F-expressing cells in blood and spleen in mouse model	
	N/A	ESSENTIAL (phase II clinical trial): ongoing; Assessment of safety and efficacy of selinexor in MF patients who are refractory and intolerant to JAK1/2 inhibitors		[336]
CDK6 inhibitor	N/A	Overexpressed CDK6 in MPN increases chronic inflammation - CDK6 in MPN binds to NF-κB p65 subunit to increase NF-κB pathway and IL-8 expression - CDK6 reduces the transcription of genes that encode IkB, leading to NF-κB activation to drive chronic inflammation Inhibition of CDK6 reduces inflammation	Absence of CDK6 decreased spleen size and induced symptom relief in murine modelsReduced pro-inflammatory cytokines in plasma of *JAK2*V617F/CDK6^−/−^ murine modelsPalbociclib is a CDK4/6 inhibitor that decreased splenomegaly in MPL mouse models, synergistic effect was seen when co-treated with ruxolitinib	[337,338,339]
		Promotion of quiescence and deactivation of *JAK2*V617F MPN stem cells	*JAK2*V617F/CDK6^−/−^ murine models had elevated short term LT-HSCs	
		Apoptosis	Palbociclib drove apoptosis in JAK2V617F haematopoietic cells in combination with ruxolitinib	
Reparixin	N/A	Reparixin inhibits IL-8 receptor and reduces proliferation of splenic endothelial cells, leading to a disrupted niche that cannot sustain MF HSC growth	MF spleen showed elevated LCN2 levels that increased MF haematopoiesis Reparixin led to the reduction of MF CD34^+^ cells by 35%	[14,268,340]

N/A: not applicable (used as single agent); ATO: arsenic trioxide; IFNα: interferon-alpha; PML: promyelocytic leukemia protein; NB: nuclear bodies; LSC: leukemic stem cells; BFU-E: burst-forming unit-erythroid; WT: wild type; AMPK: AMP-activated protein kinase; mTOR: mammalian target of rapamycin; Mcl-1: myeloid cell leukemia 1; SHP2: SH2 containing protein tyrosine phosphatase-2; ROS: reactive oxygen species; PP2A: protein phosphatase 2; PCR: polymerase chain reaction; BM: bone marrow; XPO1: exportin 1; SINE: selective inhibitors of nuclear export; NCT: nuclear cytoplasmic transport; NF-κB: nuclear factor kappa-light-chain-enhancer of activated B cells; IL-8: interleukin-8; IkB: inhibitor of NF-κB protein; LT-HSC: long term hematopoietic stem cell; LCN2: lipocalin-2.

## Data Availability

Not applicable.

## Supplementary Materials

The following are available online at https://www.mdpi.com/1422-0067/22/2/659/s1, File S1: Prognostic models for myelofibrosis. File S2: Molecular pathways in chronic myeloid leukemia and their therapeutic targeting. File S3: The bone marrow microenvironment in chronic myeloid leukemia and its therapeutic targets. File S4: Immunologic pathways and their therapeutic targeting in chronic myeloid leukemia. File S5: Molecular pathways in Philadelphia chromosome-negative myeloproliferative neoplasms and their therapeutic targeting. File S6: The bone marrow microenvironment in Philadelphia chromosome-negative myeloproliferative neoplasms and its therapeutic targeting. File S7: Immunologic pathways and their therapeutic targeting in Philadelphia chromosome-negative myeloproliferative neoplasms. File S8: Legends and abbreviations for Files S2–S7.
