# Supplementary material for: Targeting Abnormal Hematopoietic Stem Cells in Chronic Myeloid Leukemia and Philadelphia Chromosome-Negative Classical Myeloproliferative Neoplasms
Source: Int J Mol Sci. 2021 Jan 11;22(2):659. doi: 10.3390/ijms22020659 (PMC7827471; doi:10.3390/ijms22020659)
Supplement: Supplementary file 1 [file ijms-22-00659-s001.pdf]

Supplemental file 1: Prognostic models for myelofibrosis

Prognostic models for primary myelofibrosis [1-5]

Variable	IPSS	DIPSS	DIPSS-plus
**Age > 65**	1	1	1
**Constitutional Symptoms**	1	1	1
**Hb < 10g/dL**	1	2	1
**WBC > 25 x 10^9/L**	1	1	1
**PB blasts 1% or more**	1	1	1
**Platelet < 100 x 10^9/L**	-	-	1
**RBC transfusion need**	-	-	1
**Unfavourable karyotype[A]**	-	-	1

IPSS: international prognostic scoring system; DIPSS: dynamic international prognostic scoring system; Hb: Haemoglobin; WBC: white blood cell; PB: peripheral; RBC: red blood cell.

A: Unfavourable karyotype comprised +8, -7/7q-, i(17q), inv(3), -5/5q-, 12p- and 11q23 rearrangements

Overall survivals predicted by prognostic models in primary myelofibrosis [1-5]

Prognostic System	Risk	Score	Median OS (months)
**Lille score[A]**	Low	0	93
	Intermediate	1	26
	High	2	13
**IPSS**	Low	0	135
	Int-1	1	95
	Int-2	2	48
	High	$\geq$3	27
**DIPSS**	Low	0	NR
	Int-1	1-2	170
	Int-2	3-4	48
	High	5-6	18
**DIPSS-plus**	Low	0	185
	Int-1	1	78
	Int-2	2-3	35
	High	4-6	16

IPSS: international prognostic scoring system; DIPSS: dynamic international prognostic scoring system; OS: overall survival

A: Haemoglobin <10 g/dL and leucocyte count < 4 or > 30 x 10^9 are the two adverse prognostic indicators with a score of 1 for each.

Prognostic models integrating clinical risk variable, cytogenetics and gene mutations in PMF [6-14]

Variable	MIPSS70	MIPSS70+	MIPSS70+ v2.0	GIPSS
**Anaemia**	1 (Hb <10g/dL)	1 (Hb < 10g/dL)	2 (Hb < 9g/dL in men, < 8g/dL in women)<br>1 (Hb 9-10.9g/dL in men, 8-9.9g/dL in women)	
**WBC > 25 x 10^9/L**	2	-	-	-
**Platelet < 100 x 10^9/L**	2	-	-	-
**Circulating blasts ≥ 2%**	1	1	1	-
**BM fibrosis grade ≥ 2**	1	-	-	-
**Constitutional symptoms**	1	1	2	-
**Absence of type 1/type 1-like CALR mutations**	1	2	2	1
**Presence of an HMR mutation**	1	1	2	1 (*ASXL1*), 1(*SRSF2*), 1 (*U2AF1*Q157)
**Presence of ≥2 or more HMR mutations[A]**	2	2	3	-
**Unfavourable karyotype[B]**	-	3	3	1
**Very high risk karyotype[B]**	-	-	4	2

MIPSS: mutation-enhanced international prognostic scoring system for transplant-age patients; v2.0: Version 2.0; GIPSS: genetically inspired prognosic scoring system; Hb: haemoglobin; WBC: white blood cell; BM: bone marrow; HMR: high-molecular risk.

A: HMR mutations comprise *ASXL1, EZH2, IDH1/2, SRFS2* and, in addition, *U2AF1*Q157 for MIPSS70+ version 2.0.

B: Unfavourable karyotype and very high risk karyotype was defined using the revised cytogenetic risk stratification for primary myelofibrosis [13]

Overall survival predicted by prognostic models integrating gene mutations in PMF [6-14]

Prognostic System	Risk	Score	Median OS (years)
**MIPSS70 (3-tiered)**	Low	0-1	Not reached
	Intermediate	2-4	6.3
	High	≥5	3.1
**MIPSS70-plus *version 2.0***	Very low	0	Not reached
**(5-tiered)**	Low	1-2	16.4
	Intermediate	3-4	7.7
	High	5-8	4.8
	Very high	≥9	1.8
**GIPSS (4-tiered)**	Low	0	26.4
	Int-1	1	8
	Int-2	2	4.2
	High	≥3	2

MIPSS: mutation-enhanced international prognostic scoring system for transplant-age patients; v2.0: Version 2.0; GIPSS: genetically inspired prognostic scoring system; OS: overall survival

References:

1. Cervantes, F.; Dupriez, B.; Pereira, A.; Passamonti, F.; Reilly, J. T.; Morra, E.; Vannucchi, A. M.; Mesa, R. A.; Demory, J. L.; Barosi, G.; Rumi, E.; Tefferi, A., New prognostic scoring system for primary myelofibrosis based on a study of the International Working Group for Myelofibrosis Research and Treatment. *Blood* **2009,** 113, (13), 2895-901.
2. Passamonti, F.; Cervantes, F.; Vannucchi, A. M.; Morra, E.; Rumi, E.; Pereira, A.; Guglielmelli, P.; Pungolino, E.; Caramella, M.; Maffioli, M.; Pascutto, C.; Lazzarino, M.; Cazzola, M.; Tefferi, A., A dynamic prognostic model to predict survival in primary myelofibrosis: a study by the IWG-MRT (International Working Group for Myeloproliferative Neoplasms Research and Treatment). *Blood* **2010,** 115, (9), 1703-8.
3. Gangat, N.; Caramazza, D.; Vaidya, R.; George, G.; Begna, K.; Schwager, S.; Van Dyke, D.; Hanson, C.; Wu, W.; Pardanani, A.; Cervantes, F.; Passamonti, F.; Tefferi, A., DIPSS plus: a refined Dynamic International Prognostic Scoring System for primary myelofibrosis that incorporates prognostic information from karyotype, platelet count, and transfusion status. *J Clin Oncol* **2011,** 29, (4), 392-7.
4. Morel, P.; Duhamel, A.; Hivert, B.; Stalniekiewicz, L.; Demory, J. L.; Dupriez, B., Identification during the follow-up of time-dependent prognostic factors for the competing risks of death and blast phase in primary myelofibrosis: a study of 172 patients. *Blood* **2010,** 115, (22), 4350-5.

5. Rumi, E.; Cazzola, M., Diagnosis, risk stratification, and response evaluation in classical myeloproliferative neoplasms. *Blood* **2017,** 129, (6), 680-692.
6. Guglielmelli, P.; Lasho, T. L.; Rotunno, G.; Mudireddy, M.; Mannarelli, C.; Nicolosi, M.; Pacilli, A.; Pardanani, A.; Rumi, E.; Rosti, V.; Hanson, C. A.; Mannelli, F.; Ketterling, R. P.; Gangat, N.; Rambaldi, A.; Passamonti, F.; Barosi, G.; Barbui, T.; Cazzola, M.; Vannucchi, A. M.; Tefferi, A., MIPSS70: Mutation-Enhanced International Prognostic Score System for Transplantation-Age Patients With Primary Myelofibrosis. *J Clin Oncol* **2018,** 36, (4), 310-318.
7. Guglielmelli, P.; Lasho, T. L.; Rotunno, G.; Score, J.; Mannarelli, C.; Pancrazzi, A.; Biamonte, F.; Pardanani, A.; Zoi, K.; Reiter, A.; Duncombe, A.; Fanelli, T.; Pietra, D.; Rumi, E.; Finke, C.; Gangat, N.; Ketterling, R. P.; Knudson, R. A.; Hanson, C. A.; Bosi, A.; Pereira, A.; Manfredini, R.; Cervantes, F.; Barosi, G.; Cazzola, M.; Cross, N. C.; Vannucchi, A. M.; Tefferi, A., The number of prognostically detrimental mutations and prognosis in primary myelofibrosis: an international study of 797 patients. *Leukemia* **2014,** 28, (9), 1804-10.
8. Tefferi, A., Primary myelofibrosis: 2019 update on diagnosis, risk-stratification and management. *American journal of hematology* **2018,** 93, (12), 1551-1560.
9. Rozovski, U.; Verstovsek, S.; Manshouri, T.; Dembitz, V.; Bozinovic, K.; Newberry, K.; Zhang, Y.; Bove, J. E. t.; Pierce, S.; Kantarjian, H.; Estrov, Z., An accurate, simple prognostic model consisting of age, JAK2, CALR, and MPL mutation status for patients with primary myelofibrosis. *Haematologica* **2017,** 102, (1), 79-84.
10. Rumi, E.; Pietra, D.; Pascutto, C.; Guglielmelli, P.; Martinez-Trillos, A.; Casetti, I.; Colomer, D.; Pieri, L.; Pratcorona, M.; Rotunno, G.; Sant'Antonio, E.; Bellini, M.; Cavalloni, C.; Mannarelli, C.; Milanesi, C.; Boveri, E.; Ferretti, V.; Astori, C.; Rosti, V.; Cervantes, F.; Barosi, G.; Vannucchi, A. M.; Cazzola, M.; Associazione Italiana per la Ricerca sul Cancro Gruppo Italiano Malattie Mieloproliferative, I., Clinical effect of driver mutations of JAK2, CALR, or MPL in primary myelofibrosis. *Blood* **2014,** 124, (7), 1062-9.
11. Tefferi, A.; Guglielmelli, P.; Nicolosi, M.; Mannelli, F.; Mudireddy, M.; Bartalucci, N.; Finke, C. M.; Lasho, T. L.; Hanson, C. A.; Ketterling, R. P.; Begna, K. H.; Naseema, G.; Pardanani, A.; Vannucchi, A. M., GIPSS: genetically inspired prognostic scoring system for primary myelofibrosis. *Leukemia* **2018,** 32, (7), 1631-1642.
12. Tefferi, A.; Guglielmelli, P.; Pardanani, A.; Vannucchi, A. M., Myelofibrosis Treatment Algorithm 2018. *Blood cancer journal* **2018,** 8, (8), 72.
13. Tefferi, A.; Nicolosi, M.; Mudireddy, M.; Lasho, T. L.; Gangat, N.; Begna, K. H.; Hanson, C. A.; Ketterling, R. P.; Pardanani, A., Revised cytogenetic risk stratification in primary myelofibrosis: analysis based on 1002 informative patients. *Leukemia* **2018,** 32, (5), 1189-1199.
14. Tefferi, A.; Guglielmelli, P.; Lasho, T. L.; Gangat, N.; Ketterling, R. P.; Pardanani, A.; Vannucchi, A. M., MIPSS70+ Version 2.0: Mutation and Karyotype-Enhanced International Prognostic Scoring System for Primary Myelofibrosis. *J Clin Oncol* **2018,** 36, (17), 1769-1770.

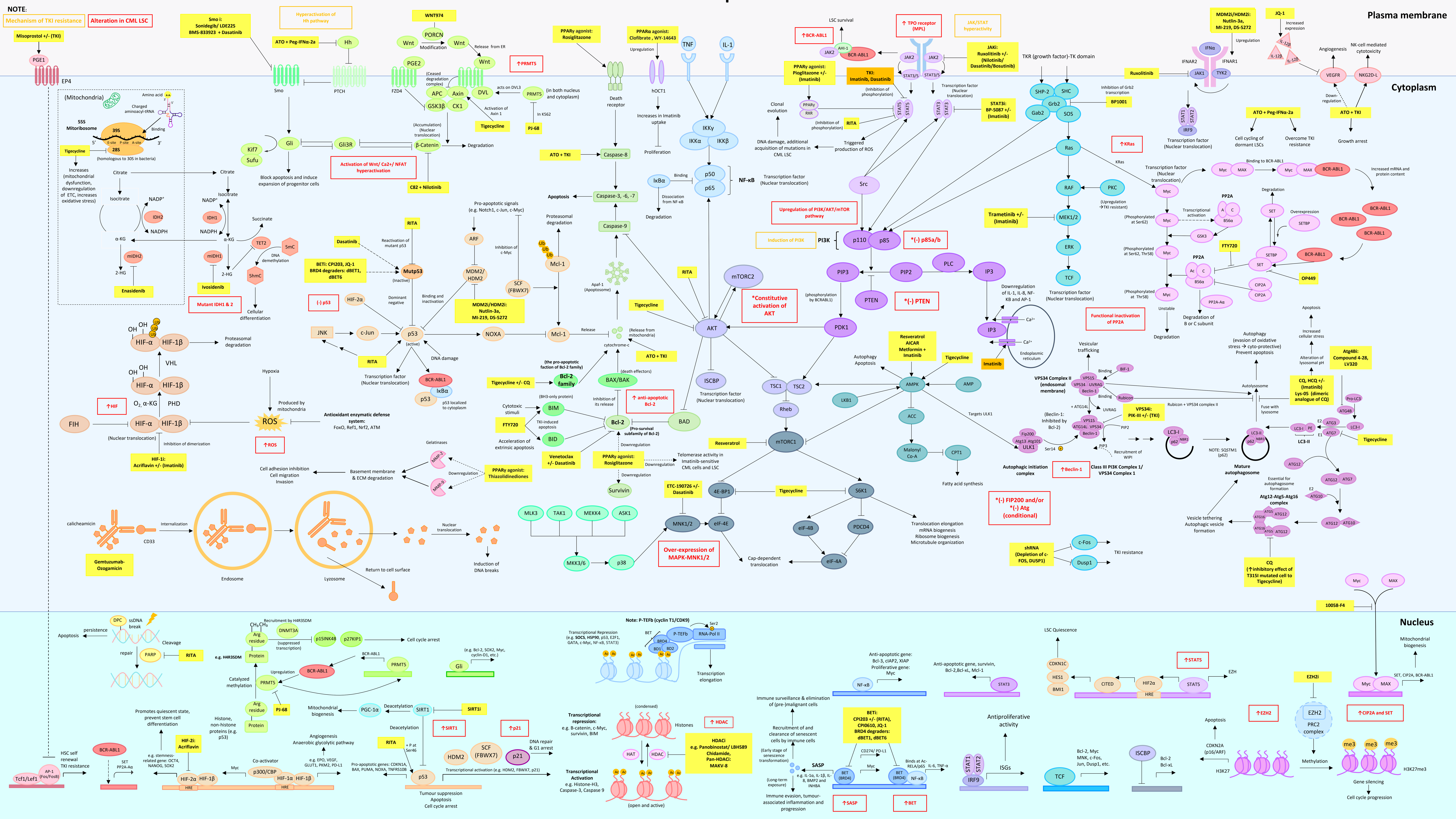
NOTE:
Mechanism of TKI resistance
Alteration in CML LSC
Plasma membrane
Cytoplasm
Nucleus
Misoprostol +/- (TKI)
PGE1
EP4
(Mitochondria)
Amino acid
a.a.
Charged aminoacyl-tRNA
55S Mitoribosome
39S
28S
E-site P-site A-site
Binding
(homologous to 30S in bacteria)
Tigecycline
Increases (mitochondrial dysfunction, downregulation of ETC, increases oxidative stress)
Citrate
Isocitrate
NADP⁺
NADPH
IDH2
IDH1
α-KG
2-HG
mIDH2
mIDH1
Succinate
TET2
5mC
DNA demethylation
5hmC
Cellular differentiation
Enasidenib
Ivosidenib
Mutant IDH1 & 2
Smo i: Sonidegib/ LDE225 BMS-833923 + Dasatinib
Hyperactivation of Hh pathway
ATO + Peg-IFNα-2a
Hh
Smo
PTCH
Gli
Gli3R
Kif7
Sufu
Block apoptosis and induce expansion of progenitor cells
WNT974
PORCN
Wnt
Modification
Release from ER
PGE2
FZD4
(Ceased degradation complex)
APC
Axin
DVL
GSK3β
CK1
acts on DVL3
PRMT5
(in both nucleus and cytoplasm)
In K562
PJ-68
Activation of Axin 1
Tigecycline
↑PRMT5
(Accumulation) (Nuclear translocation)
β-Catenin
Degradation
Activation of Wnt/ Ca2+/ NFAT hyperactivation
C82 + Nilotinib
PPARγ agonist: Rosiglitazone
Death receptor
ATO + TKI
Caspase-8
Caspase-3, -6, -7
Apoptosis
Caspase-9
PPARα agonist: Clofibrate , WY-14643
Upregulation
hOCT1
Increases in Imatinib uptake
Proliferation
TNF
IL-1
IKKγ
IKKα
IKKβ
IκBα
Binding
Dissociation from NF-κB
Degradation
p50
p65
NF-κB
Transcription factor (Nuclear translocation)
Pro-apoptotic signals (e.g. Notch1, c-Jun, c-Myc)
ARF
MDM2/ HDM2
Inhibition of c-Myc
SCF (FBXW7)
Proteasomal degradation
Ub
Mcl-1
RITA
Dasatinib
Reactivation of mutant p53
Mutp53
(Inactive)
BETi: CPI203, JQ-1 BRD4 degraders: dBET1, dBET6
(-) p53
HIF-2α
Dominant negative
Binding and inactivation
MDM2i/HDM2i: Nutlin-3a, MI-219, DS-5272
JNK
c-Jun
p53
(active)
NOXA
Release
(Release from mitochondria)
cytochrome-c
Apaf-1 (Apoptosome)
Tigecycline
ATO + TKI
DNA damage
Transcription factor (Nuclear translocation)
BCR-ABL1
IκBα
p53 localized to cytoplasm
(the pro-apoptotic faction of Bcl-2 family)
Bcl-2 family
Tigecycline +/- CQ
(death effectors)
BAX/BAK
Inhibition of its release
↑ anti-apoptotic Bcl-2
(BH3-only protein)
BIM
BID
Cytotoxic stimuli
TKI-induced apoptosis
FTY720
Acceleration of extrinsic apoptosis
Bcl-2
(Pro-survival subfamily of Bcl-2)
BAD
Venetoclax +/- Dasatinib
Downregulation
PPARγ agonist: Rosiglitazone
Telomerase activity in Imatinib-sensitive CML cells and LSC
Survivin
OH
HIF-α
HIF-1β
Proteasomal degradation
VHL
PHD
O2, α-KG
↑HIF
FIH
(Nuclear translocation)
Inhibition of dimerization
HIF-1i: Acriflavin +/- (Imatinib)
Hypoxia
Produced by mitochondria
ROS
↑ROS
Antioxidant enzymatic defense system: FoxO, Ref1, Nrf2, ATM
Gelatinases
MMP-2
MMP-9
Downregulation
PPARγ agonist: Thiazolidinediones
Basement membrane & ECM degradation
Cell adhesion inhibition Cell migration Invasion
calicheamicin
CD33
Internalization
Gemtuzumab-Ozogamicin
Endosome
Lyzosome
Nuclear translocation
Induction of DNA breaks
Return to cell surface
MLK3
TAK1
MEKK4
ASK1
MKK3/6
p38
MNK1/2
ETC-190726 +/- Dasatinib
Over-expression of MAPK-MNK1/2
eIF-4E
4E-BP1
Cap-dependent translocation
mTORC2
RITA
*Constitutive activation of AKT
AKT
ISCBP
Transcription factor (Nuclear translocation)
TSC1
TSC2
Rheb
mTORC1
Resveratrol
Tigecycline
S6K1
eIF-4B
PDCD4
eIF-4A
Translocation elongation mRNA biogenesis Ribosome biogenesis Microtubule organization
LSC survival
↑BCR-ABL1
AHI-1
JAK2
BCR-ABL1
↑ TPO receptor (MPL)
JAK/STAT hyperactivity
JAKi: Ruxolitinib +/- (Nilotinib/ Dasatinib/Bosutinib)
STAT3/5
PPARγ agonist: Pioglitazone +/- (Imatinib)
TKI: Imatinib, Dasatinib
(Inhibition of phosphorylation)
Transcription factor (Nuclear translocation)
STAT3i: BP-5087 +/- (Imatinib)
PPARγ
RXR
Clonal evolution
RITA
DNA damage, additional acquisition of mutations in CML LSC
Triggered production of ROS
Src
Upregulation of PI3K/AKT/mTOR pathway
Induction of PI3K
PI3K
p110
p85
*(-) p85a/b
PIP3
PIP2
PLC
IP3
PTEN
*(-) PTEN
(phosphorylation by BCRABL1)
PDK1
Downregulation of IL-1, IL-8, NF-KB and AP-1
Ca²⁺
Endoplasmic reticulum
Imatinib
Resveratrol AICAR Metformin + Imatinib
Autophagy Apoptosis
AMPK
AMP
LKB1
ACC
Malonyl Co-A
CPT1
Fatty acid synthesis
Targets ULK1
Fip200
Atg13 Atg101
ULK1
Ser14
Autophagic initiation complex
↑Beclin-1
*(-) FIP200 and/or *(-) Atg (conditional)
TKR (growth factor)-TK domain
SHP-2
SHC
Grb2
Gab2
SOS
Inhibition of Grb2 transcription
BP1001
Ras
↑KRas
KRas
RAF
PKC
(Upregulation →TKI resistant)
Trametinib +/- (Imatinib)
MEK1/2
ERK
TCF
Transcription factor (Nuclear translocation)
Functional inactivation of PP2A
shRNA (Depletion of c-FOS, DUSP1)
c-Fos
Dusp1
TKI resistance
MDM2i/HDM2i: Nutlin-3a, MI-219, DS-5272
Upregulation
IFNα
IFNAR2
IFNAR1
Ruxolitinib
JAK1
TYK2
STAT1
STAT2
IRF9
Transcription factor (Nuclear translocation)
JQ-1
Increased expression
IL-12β
Angiogenesis
NK-cell mediated cytotoxicity
VEGFR
NKG2D-L
Down-regulation
ATO + TKI
Growth arrest
ATO + Peg-IFNα-2a
Cell cycling of dormant LSCs
Overcome TKI resistance
Transcription factor (Nuclear translocation)
Myc
MAX
Binding to BCR-ABL1
Increased mRNA and protein content
(Phosphorylated at Ser62)
Transcriptional activation
GSK3
(Phosphorylated at Ser62, Thr58)
(Phosphorylated at Thr58)
Unstable
Degradation
PP2A
A
C
B56α
Degradation
SET
Overexpression
SETBP
FTY720
OP449
CIP2A
PP2A-Aα
Degradation of B or C subunit
Apoptosis
Increased cellular stress
Autophagy (evasion of oxidative stress → cyto-protective) Prevent apoptosis
Alteration of lysosomal pH
Atg4Bi: Compound 4-28, LV320
CQ, HCQ +/- (Imatinib) Lys-05 (dimeric analogue of CQ)
Vesicular trafficking
Binding
Bif-1
VPS34 Complex II (endosomal membrane)
VPS15
VPS34
UVRAG
Beclin-1
Rubicon
Rubicon + VPS34 complex II
(Beclin-1: Inhibited by Bcl-2)
VPS34i: PIK-III +/- (TKI)
ATG14L
PIP2
PIP3
Recruitment of WIPI
Class III PI3K Complex 1/ VPS34 Complex 1
LC3-I
p62
NBR1
NOTE: SQSTM1 (p62)
LC3-II
Autolysosome
Fuse with lysosome
Mature autophagosome
Pro-LC3
ATG4B
ATG3
ATG7
PE
Tigecycline
ATG12
ATG10
ATG5
ATG16
Essential for autophagosome formation
Atg12-Atg5-Atg16 complex
Vesicle tethering Autophagic vesicle formation
CQ (↑inhibitory effect of T315I mutated cell to Tigecycline)
10058-F4
DPC
ssDNA break
persistence
Apoptosis
Cleavage
repair
PARP
RITA
Recruitment by H4R3SDM
DNMT3A
Arg residue
(suppressed transcription)
p15INK4B
p27KIP1
Cell cycle arrest
e.g. H4R3SDM
Protein
BCR-ABL1
PRMT5
Catalyzed methylation
Upregulation
PJ-68
Gli
(e.g. Bcl-2, SOX2, Myc, cyclin-D1, etc.)
Histone, non-histone proteins (e.g. p53)
Mitochondrial biogenesis
PGC-1α
Deacetylation
SIRT1
SIRT1i
↑SIRT1
↑p21
RITA
+ P at Ser46
Promotes quiescent state, prevent stem cell differentiation
HIF-2i: Acriflavin
e.g. stemness-related gene: OCT4, NANOG, SOX2
Myc
Co-activator
p300/CBP
HIF-2α
HIF-1β
HIF-1α
HRE
Angiogenesis Anaerobic glycolytic pathway
e.g. EPO, VEGF, GLUT1, PKM2, PD-L1
Pro-apoptotic genes: CDKN1A, BAX, PUMA, NOXA, TNFRS10B
HDM2
SCF (FBXW7)
p21
DNA repair & G1 arrest
Transcriptional activation (e.g. HDM2, FBWX7, p21)
Tumour suppression Apoptosis Cell cycle arrest
HSC self renewal TKI resistance
AP-1 (Fos/FosB)
Tcf1/Lef1
SET PP2A-Aα
Note: P-TEFb (cyclin T1/CDK9)
Transcriptional Repression (e.g. SOCS, HSP90, p53, E2F1, GATA, c-Myc, NF-κB, STAT3)
BET
BRD4
BD1
BD2
P-TEFb
RNA-Pol II
Ser2
Transcription elongation
Transcriptional repression: e.g. B-catenin, c-Myc, survivin, BIM
(condensed)
Histones
↑ HDAC
HAT
HDAC
HDACi: e.g. Panobinostat/ LBH589 Chidamide, Pan-HDACi: MAKV-8
Transcriptional Activation e.g. Histone-H3, Caspase-3, Caspase 9
(open and active)
Anti-apoptotic gene: Bcl-3, cIAP2, XIAP Proliferative gene: Myc
NF-κB
Anti-apoptotic gene, survivin, Bcl-2,Bcl-xL, Mcl-1
STAT3
Immune surveillance & elimination of (pre-)malignant cells
Recruitment of and clearance of senescent cells by immune cells
BETi: CPI203 +/- (RITA), CPI0610, JQ-1 BRD4 degraders: dBET1, dBET6
CD274/ PD-L1
Myc
Binds at Ac-RELA/p65
IL-6, TNF-α
(Early stage of senescence transformation)
SASP
(Long-term exposure)
e.g. IL-1α, IL-1β, IL-8, BMP2 and INHBA
BET (BRD4)
Immune evasion, tumour-associated inflammation and progression
↑SASP
↑BET
Antiproliferative activity
ISGs
IRF9
TCF
Bcl-2, Myc MNK, c-Fos, Jun, Dusp1, etc.
ISCBP
Bcl-2 Bcl-xL
LSC Quiescence
CDKN1C
HES1
BMI1
CITED
HIF2α
HRE
STAT5
↑STAT5
EZH
EZH2i
EZH2
PRC2 complex
↑EZH2
Methylation
Apoptosis
CDKN2A (p16/ARF)
H3K27
me3
H3K27me3
Gene silencing
Cell cycle progression
↑CIP2A and SET
Mitochondrial biogenesis
MAX
SET, CIP2A, BCR-ABL1

Bone marrow microenvironment in CML

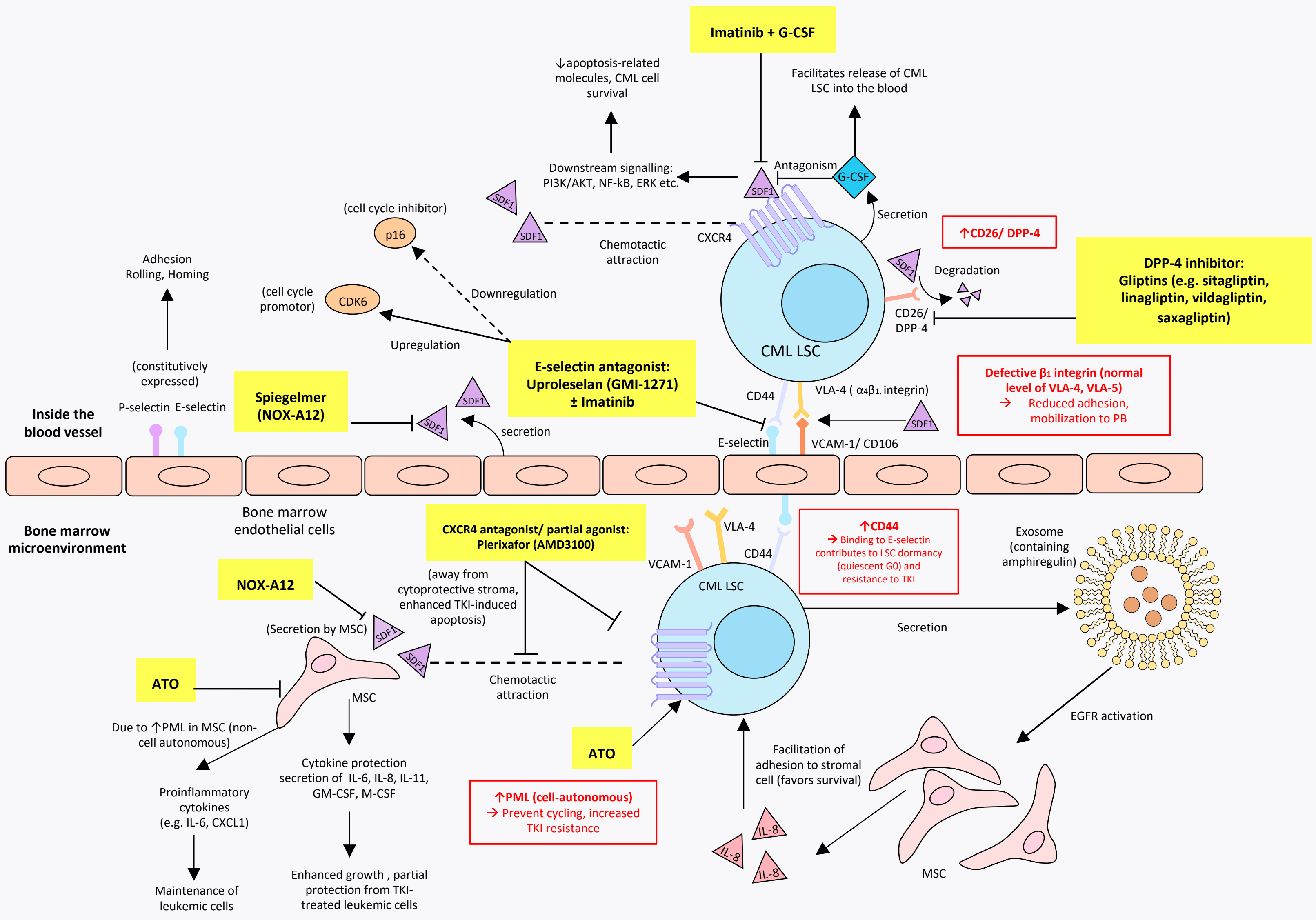

Immunotherapy in CML

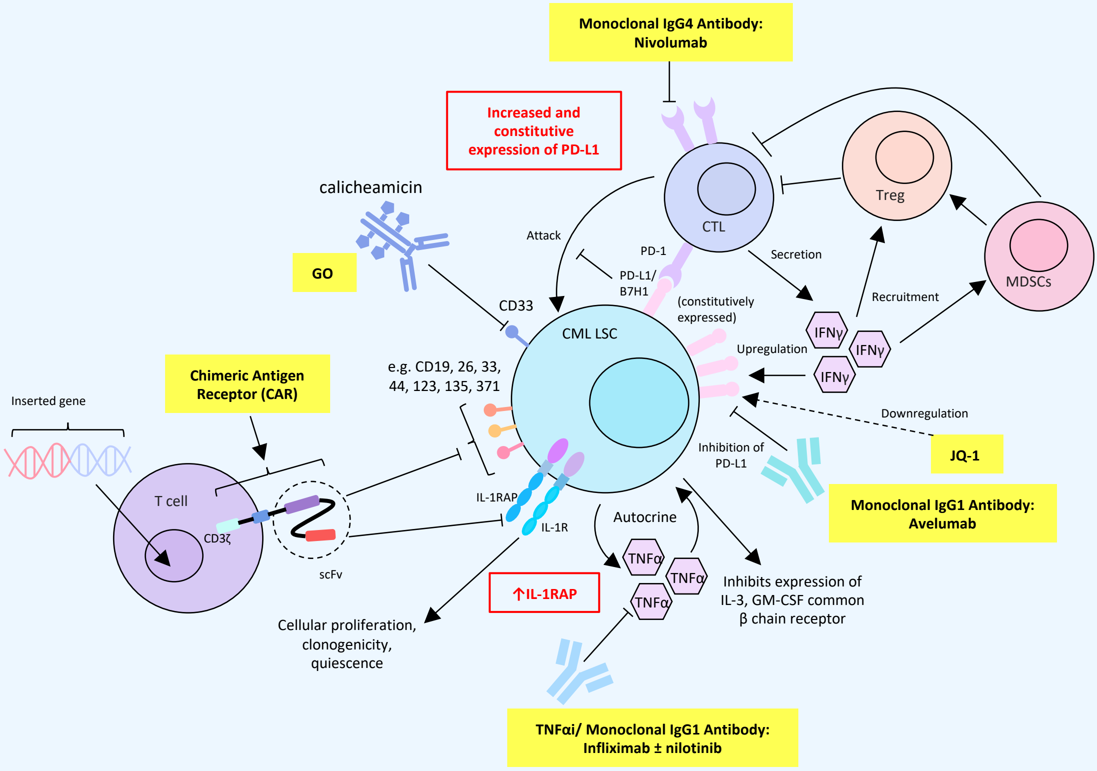

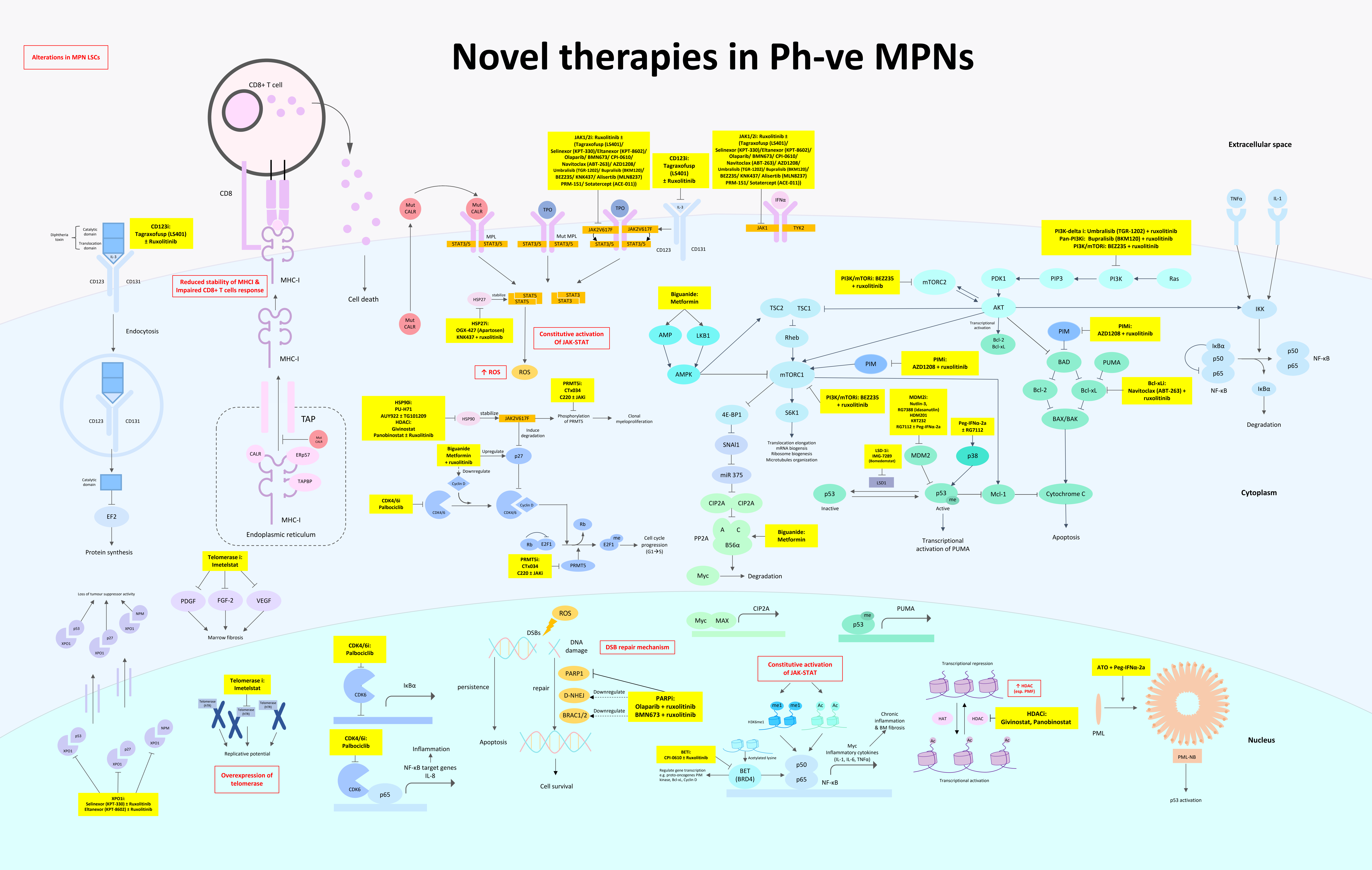
Novel therapies in Ph-ve MPNs
Alterations in MPN LSCs
CD8+ T cell
CD8
MHC-I
Cell death
Reduced stability of MHCI & Impaired CD8+ T cells response
TAP
CALR
ERp57
TAPBP
Endoplasmic reticulum
Diphtheria toxin
Catalytic domain
Translocation domain
IL-3
CD123i: Tagraxofusp (LS401) ± Ruxolitinib
CD123
CD131
Endocytosis
EF2
Protein synthesis
Mut CALR
MPL
Mut MPL
TPO
STAT3/5
JAK2V617F
JAK1/2i: Ruxolitinib ± (Tagraxofusp (LS401)/ Selinexor (KPT-330)/Eltanexor (KPT-8602)/ Olaparib/ BMN673/ CPI-0610/ Navitoclax (ABT-263)/ AZD1208/ Umbralisib (TGR-1202)/ Bupralisib (BKM120)/ BEZ235/ KNK437/ Alisertib (MLN8237) PRM-151/ Sotatercept (ACE-011))
CD123i: Tagraxofusp (LS401) ± Ruxolitinib
IFNα
JAK1
TYK2
HSP27
stabilize
STAT5
STAT3
HSP27i: OGX-427 (Apartosen) KNK437 + ruxolitinib
Constitutive activation Of JAK-STAT
↑ ROS
ROS
HSP90i: PU-H71 AUY922 ± TG101209 HDACi: Givinostat Panobinostat ± Ruxolitinib
HSP90
PRMT5i: CTx034 C220 ± JAKi
Phosphorylation of PRMT5
Clonal myeloproliferation
Induce degradation
Biguanide Metformin + ruxolitinib
Upregulate
Downregulate
p27
CDK4/6i Palbociclib
CDK4/6
Cyclin D
Rb
E2F1
me
Cell cycle progression (G1→S)
PRMT5
Telomerase i: Imetelstat
PDGF
FGF-2
VEGF
Marrow fibrosis
Loss of tumour suppressor activity
p53
p27
NPM
XPO1
XPO1i: Selinexor (KPT-330) ± Ruxolitinib Eltanexor (KPT-8602) ± Ruxolitinib
Telomerase (hTR)
Replicative potential
Overexpression of telomerase
CDK4/6i: Palbociclib
CDK6
IκBα
Inflammation
NF-κB target genes IL-8
p65
ROS
DSBs
DNA damage
DSB repair mechanism
persistence
repair
Apoptosis
PARP1
D-NHEJ
BRAC1/2
PARPi: Olaparib + ruxolitinib BMN673 + ruxolitinib
Cell survival
Biguanide: Metformin
AMP
LKB1
AMPK
TSC2
TSC1
Rheb
mTORC1
mTORC2
PIM
PIMi: AZD1208 + ruxolitinib
PI3K/mTORi: BEZ235 + ruxolitinib
4E-BP1
S6K1
Translocation elongation mRNA biogenesis Ribosome biogenesis Microtubules organization
SNAI1
miR 375
CIP2A
PP2A
A
C
B56α
Biguanide: Metformin
Myc
Degradation
PDK1
PIP3
PI3K
Ras
PI3K-delta i: Umbralisib (TGR-1202) + ruxolitinib Pan-PI3Ki: Bupralisib (BKM120) + ruxolitinib PI3K/mTORi: BEZ235 + ruxolitinib
AKT
Transcriptional activation
Bcl-2 Bcl-xL
BAD
PUMA
Bcl-2
Bcl-xL
Bcl-xLi: Navitoclax (ABT-263) + ruxolitinib
BAX/BAK
Cytochrome C
Apoptosis
MDM2i: Nutlin-3, RG7388 (idasanutlin) HDM201 KRT232 RG7112 ± Peg-IFNα-2a
Peg-IFNα-2a ± RG7112
MDM2
p38
LSD-1i: IMG-7289 (Bomedemstat)
LSD1
p53
Inactive
Active
Mcl-1
Transcriptional activation of PUMA
Extracellular space
TNFα
IL-1
IKK
IκBα
p50
p65
NF-κB
Degradation
Cytoplasm
Myc
MAX
CIP2A
PUMA
Constitutive activation of JAK-STAT
me1
Ac
H3K6me1
Acetylated lysine
BETi: CPI-0610 ± Ruxolitinib
BET (BRD4)
Regulate gene transcription e.g. proto-oncogenes PIM kinase, Bcl-xL, Cyclin D
NF-κB
Myc Inflammatory cytokines (IL-1, IL-6, TNFα)
Chronic inflammation & BM fibrosis
Transcriptional repression
↑ HDAC (esp. PMF)
HAT
HDAC
HDACi: Givinostat, Panobinostat
Transcriptional activation
ATO + Peg-IFNα-2a
PML
PML-NB
p53 activation
Nucleus

Bone marrow microenvironment in MPN

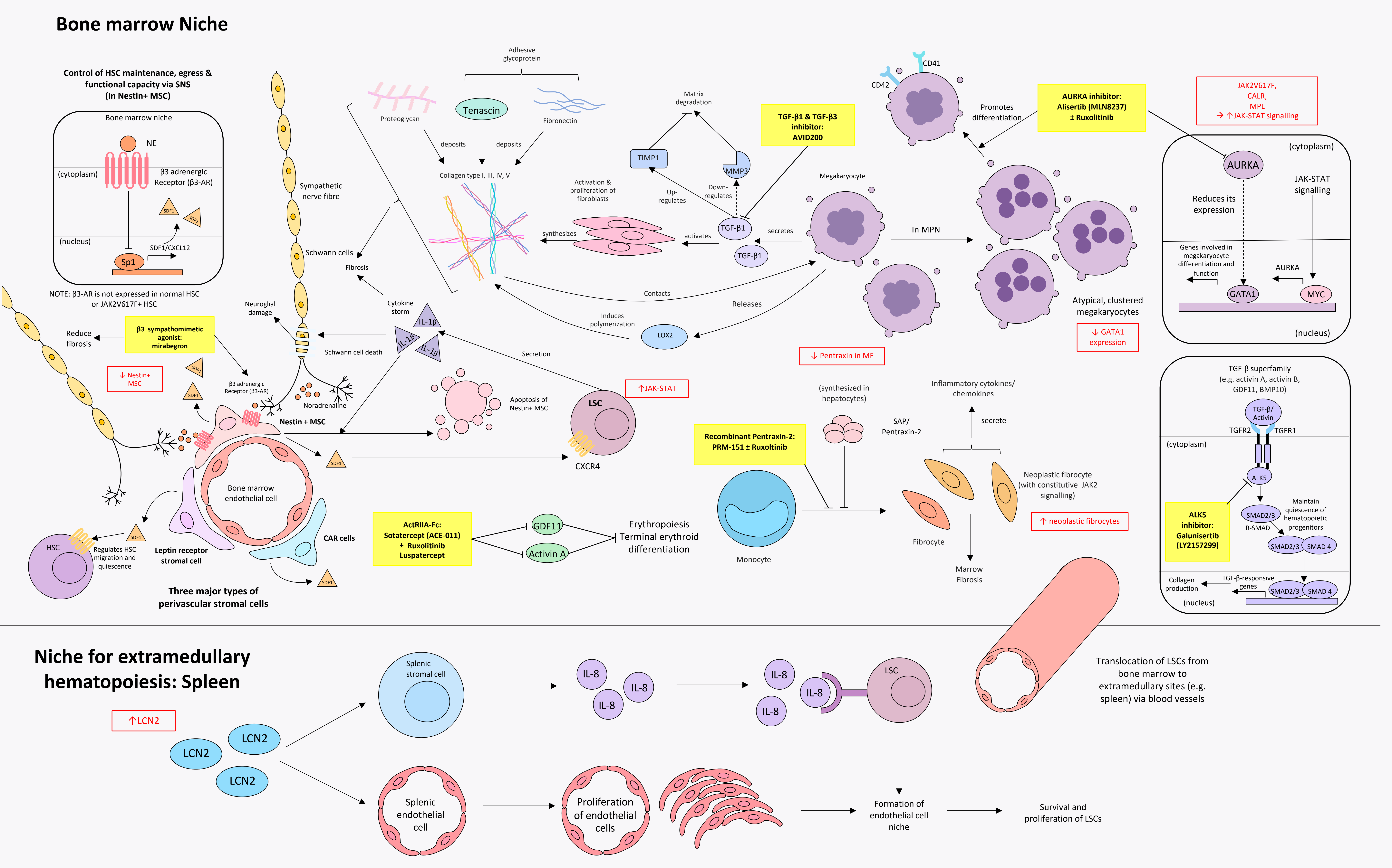

Immunotherapy in Ph-ve MPNs

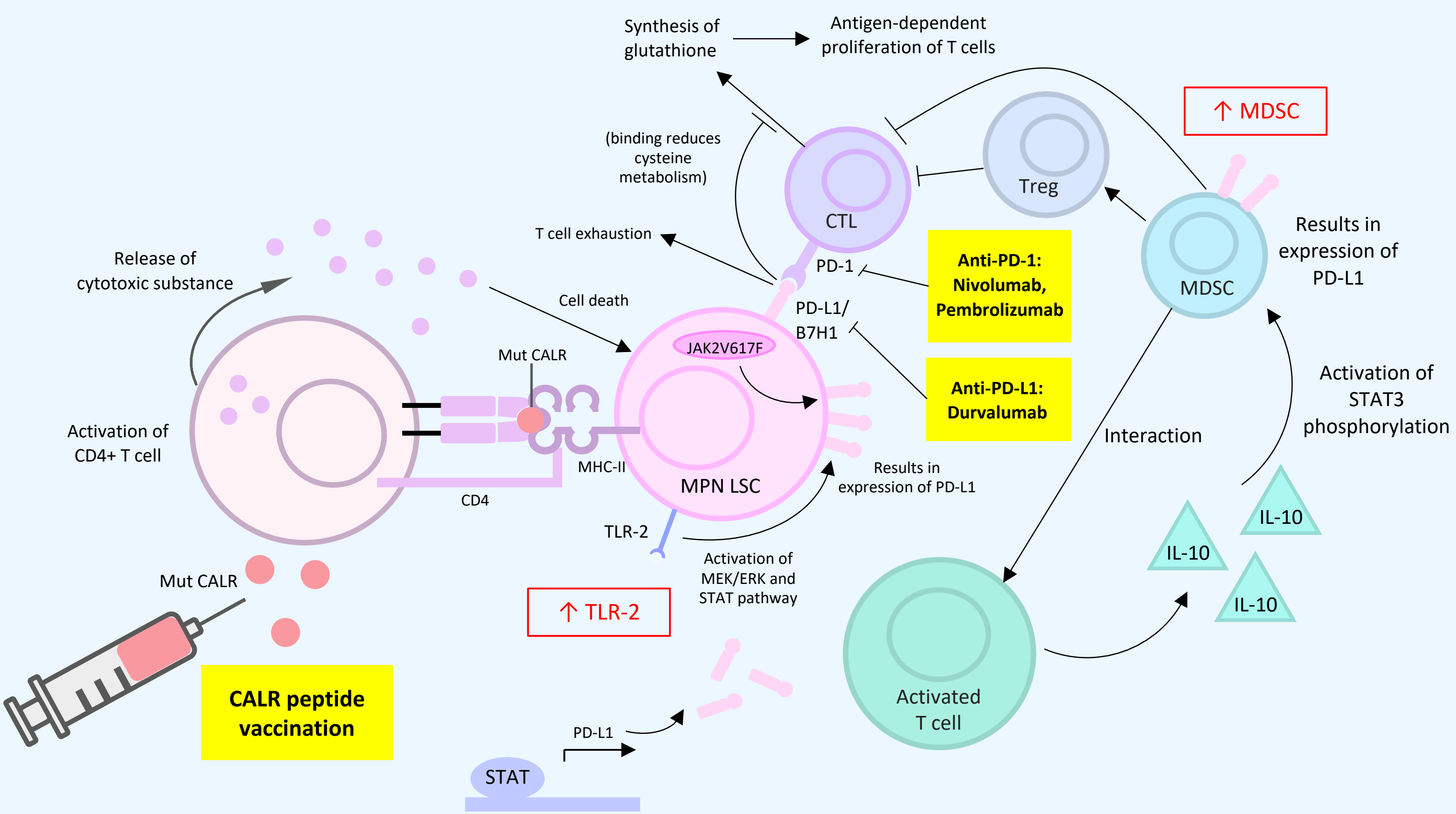

Supplemental file 2. Pathogenetic pathways in chronic myeloid leukemia and their therapeutic targeting.

Abbreviations: 2-HG, 2-hydroxyglutarate; 28S, 28S ribosomal subunit; 30S, 30S ribosomal subunit; 39S, 39S ribosomal subunit; 4E-BP1, Eukaryotic translation initiation factor 4E-binding protein 1; 5hmC, 5-hydroxymethylcytosine; 5mC, 5-methylcytosine; Ac, Acetylation; ACC, Acetyl-CoA carboxylase; AHI-1, Abelson Helper Integration Site 1; AICAR, 5-aminoimidazole-4-carboxamide riboside; AKT, Protein kinase B; AMP, adenosine monophosphate; AMPK, 5' adenosine monophosphate-activated protein kinase; AP-1, Activator protein 1; Apaf-1, Apoptotic protease activating factor 1; APC, Adenomatous polyposis coli protein; ARF, Alternative reading frame; ASK1, Apoptosis signal-regulating kinase 1; Atg101, Autophagy-related protein 101; Atg13, Autophagy-related protein 13; Atg3, Autophagy-related protein 3; Atg4B, Autophagy-related protein 4B; Atg4Bi, Autophagy-related 4B cysteine peptidase inhibitor; Atg5, Autophagy-related protein 5; Atg7, Autophagy-related protein 7; Atg10, Autophagy-related protein 10; Atg12, Autophagy-related protein 12; Atg14L, Autophagy-related protein 14-like protein, also known as Barkor, Beclin 1-associated autophagy-related key regulator; Atg16, Autophagy-related protein 16; ATM, Ataxia telangiectasia mutated; ATO, Arsenic trioxide; B56α , PP2A B subunit isoform B56-alpha; BAD, Bcl-2-antagonist of cell death; BAK, Bcl-2 antagonist/killer; BAX, Bcl-2-associated X; Bcl-2, B-cell lymphoma 2; Bcl-xL, B-cell lymphoma-extra large; BCR-ABL1, Breakpoint cluster region-Abelson murine leukaemia viral oncogene homolog 1; BD1, Bromodomain 1; BD2, Bromodomain 2; BET, Bromodomain and extra-terminal protein; BETi, Bromodomain and extra-terminal protein inhibitor; BH3, Bcl-2 homology domain 3; BID, BH3 interacting-domain death agonist; BIF-1, Bax-interacting factor 1; BIM, Bcl-2-like protein 11; BMI1, B lymphoma Mo-MLV insertion region 1 homolog; BMP2, Bone morphogenetic protein 2; BRD4, Bromodomain 4; c-Fos, cellular FBJ murine osteosarcoma viral oncogene homolog; c-Myc, cellular Myelocytomatosis oncogene; CD33, Siglec-3; CDK9, Cyclin-dependent kinase 9; CDKN1A, Cyclin-dependent kinase inhibitor 1A; CDKN1C, Cyclin-dependent kinase inhibitor 1C; CDKN2A, Cyclin-dependent kinase inhibitor 2A; cIAP2, cellular inhibitor of apoptosis 2; CIP2A, Cancerous inhibitor of PP2A; CITED, CREB-binding protein/p300-interacting transactivator with Asp/Glu-rich C-terminal domain; CK1, Casein kinase 1; CML, Chronic myeloid leukaemia; CPT1, Carnitine palmitolytransferase 1; CQ, Chloroquine; DNA, Deoxyribonucleic acid; DNMT3A, DNA (cytosine-5)-methyltransferase 3A; DPC, Dusp1, Dual-specificity phosphatase 1; DVL, Dishevelled homolog; E1, Ubiquitin-activating enzyme; E2, Ubiquitin-conjugating enzyme; E2F1, E2F transcription factor 1; E3, Ubiquitin-protein ligase; eIF-4A, Eukaryotic initiation factor-4A; eIF-4B, Eukaryotic translation initiation factor 4B; eIF-4E, Eukaryotic translation initiation factor 4E; EP4, E-type prostanoid receptor 4; EPO, Erythropoietin; ERK, also known as MAPK, Mitogen-activated protein kinase; ETC, Electron transport chain; EZH2, Enhancer of zeste homologue 2; EZH2, Enhancer of zeste homologue 2 inhibitor; FBWX7, F-box/WD repeat-containing protein 7; FIH, Factor inhibiting HIF; Fip200, FAK family kinase-interacting protein of 200 kDa; Fos, FBJ murine osteosarcoma viral oncogene homolog; FosB, FBJ murine osteosarcoma viral oncogene homolog B; FoxO, Forkhead homeobox type O family; FZD4, Frizzled-4; Gab2, Grb2-associated binding protein 2; GATA, GATA-binding factor; Gli, Glioma-associated oncogene homolog Gli3R; GLUT1, Glucose transporter 1; Grb2, Growth factor receptor-bound protein 2; GSK3, Glycogen

synthase kinase 3; GSK3β, Glycogen synthase kinase 3 beta; H3K27, 27th amino acid in Histone H3; H3K27me3, H3K27 trimethylation; H4R3SDM, Symmetric di-methyl histone H4 arginine 3; HDAC, Histone deacetylase; HDACi, Histone deacetylase inhibitor; HDM2, Human double minute 2 protein; HDM2i, Human double minute 2 protein inhibitor; HES1, Hes family BHLH transcription factor 1; Hh, Hedgehog; HIF-1i, Hypoxia-inducible factor-1 inhibitor; HIF-1α, Hypoxia-inducible factor 1 alpha; HIF-1β, Hypoxia-inducible factor-1beta; HIF-1β, Hypoxia-inducible factor 1 beta; HIF-2i, Hypoxia inducible factor 2 inhibitor; HIF-2α, Hypoxia-inducible factor-2 alpha; HIF-α, Hypoxia-inducible factor-alpha; hOCT1, Human organic cation transporter type 1; HRE, hormone response element; HSP90, Heat shock protein 90; IDH1, Isocitrate dehydrogenase 1; IDH2, Isocitrate dehydrogenase 2; IDHi, Isocitrate dehydrogenase inhibitor; IFNAR1, Interferon alpha and beta receptor subunit 1; IFNAR2, Interferon alpha and beta receptor subunit 2; IKKα, IκB kinase alpha; IKKβ, IκB kinase beta; IKKγ, IκB kinase gamma; IL-1, Interleukin-1; IL-1α, Interleukin-1 alpha; IL-1β, Interleukin-1 beta; IL-8, Interleukin-8; INHBA, Inhibin, beta A; IP3, Inositol 1,4,5-trisphosphate; IRF9, Interferon Regulatory Factor 9; ISCBP, Interferon consensus sequence binding protein; ISG, IFN-stimulated gene; IκBα, inhibitor of NFκB alpha; JAK1, Janus kinase 1; JAK2, Janus kinase 2; JAKi, Janus kinase inhibitor; JNK, c-Jun N-terminal kinase; Kif7, Kinesin-like protein KIF7; KRas, Kirsten rat sarcoma viral oncogene homolog; LC3-I, Microtubule-associated protein light chain 3 I; LC3-II, Microtubule-associated protein light chain 3 II; LKB1, Liver kinase B1; LSC, Leukemic stem cell; MAX, Myc-associated factor X; Mcl-1, Induced myeloid leukaemia cell differentiation protein Mcl-1; MDM2, Mouse double minute 2 protein, also known as E3 ubiquitin-protein ligase; MDM2i, Mouse double minute 2 protein inhibitor; me3, trimethylation; MEK1/2, Mitogen-activated protein kinase kinase 1/2; MEKK4, Mitogen-activated protein kinase kinase kinase 4; mIDH1, mutant Isocitrate dehydrogenase 1; mIDH2, mutant Isocitrate dehydrogenase 2; MKK3/6, Mitogen-activated protein kinase kinase 3/6; MLK3, Mixed-lineage protein kinase 3; MMP-2, Matrix metalloproteinase 2; MMP-9, Matrix metalloproteinase 9; MNK1/2, Mitogen-activated protein kinase interacting kinase 1/2; mTORC1, Mammalian target of rapamycin complex 1; mTORC2, Mammalian target of rapamycin complex 2; Mutp53, Mutant p53; Myc, Myelocytomatosis oncogene; NADP, Nicotinamide adenine dinucleotide phosphate; NADPH, Nicotinamide adenine dinucleotide phosphate hydrogen; NBR1, Neighbour of breast cancer 1 gene; NF-κB, Nuclear factor kappa-light-chain-enhancer of activated B cells; NKG2D-L, Natural killer group 2, member D ligand; NOXA, latin for damage, also known as PMAIP1, Phorbol-12-myristate-13-acetate-induced protein 1; Nrf2, Nuclear factor erythroid-2-related factor 2; P, phosphorylation; P-TEFb, Positive transcription elongation factor b; p110, p110 catalytic subunit of PI3K; p15INK4B, Cyclin-dependent kinase 4 inhibitor B; p27KIP1, Cyclin-dependent kinase inhibitor 1B; p300/CBP, E1A binding protein p300/cAMP-response-element-binding protein-binding protein; p38, p38 mitogen-activated protein kinases; p50, p50 subunit of NF-κB; p53, p53 upregulated modulator of apoptosis; p62, also known as SQSTM1, Sequestosome 1; p65, p65 subunit of NF-κB; p85, p85 regulatory subunit of PI3K; Pan-HDACi, Pan- HDAC inhibitor; PARP, Poly (ADP-ribose) polymerase; PD-L1, Programmed death-ligand 1; PDCD4, Programmed cell death protein 4; PDK1, 3-phosphoinositide-dependent protein kinase-1; PE, phosphatidylethanolamine; Peg-IFNα-2a, Pegylated interferon alfa-2a; PGC-1α, Peroxisome proliferator-activated receptor-gamma coactivator (PGC)-1alpha; PGE1,

Prostaglandin E1; PGE2, Prostaglandin E2; PHD, Prolyl hydroxylase domain; PI3K, Phosphoinositide 3-kinase; PIP2, Phosphatidylinositol 4,5-bisphosphate; PIP3, phosphatidylinositol-3, 4, 5-triphosphate; PKC, Protein kinase C; PKM2, Tumor M2-pyruvate kinase; PLC, Phospholipase C; PMF, Primary myelofibrosis; PORCN, O-acyl transferase porcupine; PP2A, Protein phosphatase 2A; PP2A-Aα, Protein Phosphatase 2A-A alpha; PPARγ, Peroxisome proliferator-activated receptor gamma; PRC2 complex, Polycomb repressive complex 2; PRMT5, Protein arginine methyltransferase 5; Pro-LC3, Pro-microtubule-associated protein light chain 3; PTCH, Patched; PTEN, Phosphatase and tensin homolog; PUMA, p53 upregulated modulator of apoptosis; RAF, Rapidly; accelerated fibrosarcoma; Ras, Rat sarcoma viral oncogene homolog; Ref1, Redox factor-1; RelA, also known as p65; Rheb, Ras homolog enriched in brain; RNA-Pol II, RNA polymerase II; ROS, Reactive oxygen species; RXR, Retinoid X receptor; S6K1, Ribosomal protein S6 kinase beta-1; SCF, Skp1-Cullin-F-box; SET, Protein SET; SETBP, SET-binding protein; SHC, Src homology 2 domain containing transforming protein; SHP-2, SH2 domain-containing protein tyrosine phosphatase-2; SIRT1, Sirtuin 1; SIRT1i, Sirtuin 1 inhibitor; Smo, Smoothened; Smo i, Smoothened inhibitor; SOS, Son of Sevenless; SOX2, SRY-Box Transcription Factor 2; Src, SRC Proto-Oncogene, Non-Receptor Tyrosine Kinase; ssDNA, single stranded DNA; STAT1, Signal transducer and activator of transcription 1; STAT2, Signal transducer and activator of transcription 2; STAT3, Signal transducer and activator of transcription 3; STAT3i, Signal transducer and activator of transcription 3 inhibitor; STAT5, Signal transducer and activator of transcription 5; Sufu, Suppressor of Fused; TAK1, Transforming growth factor beta-activated kinase 1; TCF, Ternary complex factors; Tcf1/Lef1, T cell factor 1 and lymphoid enhancer-binding factor 1; TET2, ten-eleven translocation methylcytosine dioxygenase 2; TK, Tyrosine kinase; TKI, Tyrosine Kinase Inhibitor; TKR, Tyrosine kinase receptor; TNF-α, Tumor necrosis factor-alpha; TNFRS10B, Tumor necrosis factor receptor superfamily 10B; tRNA, Transfer ribonucleic acid; TSC1, Tuberous sclerosis complex 1; TSC2, Tuberous sclerosis complex 2; TYK2, Tyrosine kinase 2; Ub, Ubiqutin; ULK1, Unc-51 Like Autophagy Activating Kinase 1; UVRAG, UV radiation resistance-associated gene; VEGFR, Vascular endothelial growth factor receptor; VHL, von Hippel-Lindau tumor suppressor protein; VPS15, also known as PIK3R4, Phosphoinositide 3-kinase regulatory subunit 4; VPS34, Vacuolar protein sorting 34; VPS34i, Vacuolar protein sorting 34 inhibitor; WIPI, WD-repeat protein interacting with phosphoinositides; Wnt, Wingless-related integration site; XIAP, X-linked inhibitor of apoptosis; α-KG, Alpha-ketoglutarate; β-catenin, Beta-catenin

Supplemental file 3. The bone marrow microenvironment in chronic myeloid leukemia and its therapeutic targets.

Abbreviations: Akt, Protein kinase B; ATO, Arsenic trioxide; CD106, Cluster of differentiation 106; CD26, Cluster of differentiation 26; CD44, Cluster of differentiation 44; CDK6, Cyclin-dependent kinase 6; CML, Chronic myeloid leukaemia; CXCL1, C-X-C Motif Chemokine Ligand 1; CXCR4, C-X-C Motif Chemokine Ligand 4; DPP-4, Dipeptidyl-peptidase 4; EGFR, epidermal growth factor receptor; Erk, Extracellular-signal-regulated kinase; G-CSF, Granulocyte colony-stimulating factor; GM-CSF, Granulocyte-macrophage colony-stimulating factor; IL-11, Interleukin-11; IL-6, Interleukin-6; IL-8, Interleukin-8; LSC, Leukaemic stem cells; M-CSF, Macrophage colony-stimulating factor; MSC, Mesenchymal stromal cells; NF-kB, Nuclear factor kappa-light-chain-enhancer of activated B cells; PB, Peripheral blood; PI3K, Phosphoinositide 3-kinaseSDF1, stromal cell-derived factor 1; TKI, Tyrosine kinase inhibitor; VCAM-1, Vascular cell adhesion molecule 1; VLA-4, Very late antigen-4; VLA-5, Very late antigen-5

Supplemental file 4. Immunologic pathways and their therapeutic targeting in chronic myeloid leukemia.

Abbreviations: CD123, Cluster of differentiation 123; CD135, Cluster of differentiation 135; CD19, Cluster of differentiation 19; CD26, Cluster of differentiation 26; CD33, Cluster of differentiation 33; CD371, Cluster of differentiation 371; CD3ζ, CD3 zeta chain; CD44, Cluster of differentiation 44; CTL, Cytotoxic T lymphocyte; GM-CSF, Granulocyte-macrophage colony-stimulating factor; GO, Gemtuzumab Ozogamicin; IFNγ, Interferon gamma; IgG1, Immunoglobulin G1; Immunoglobulin G4; IL-1R, Interleukin-1 receptor; IL-1RAP, Interleukin-1 receptor accessory protein; IL-3, Interleukin-1; MDSCs, Myeloid derived suppressor cells; PD-1, Programmed cell death protein 1; PD-L1, Programmed death-ligand 1; scFv, Single-chain variable fragment; TNFα, Tumour necrosis factor alpha; TNFαi, Tumour necrosis factor alpha inhibitor; Treg, Regulatory T cells

Supplemental file 5. Molecular pathways in Philadelphia chromosome-negative myeloproliferative neoplasms and their therapeutic targeting.

Abbreviations: 4E- BP1, Eukaryotic translation initiation factor 4E-binding protein 1; Ac, Acetyl-group; AKT, Protein kinase B; AMP, Adenosine monophosphate; AMPK, AMP-activated protein kinase; ATO, Arsenic trioxide; B56α , PP2A B subunit isoform B56-alpha; BAD, Bcl-2-antagonist of cell death; BAK, Bcl-2-antagonist/killer 1; BAX, Apoptosis regulator BAX; Bcl-2, B-cell lymphoma 2; Bcl-xL, B-cell lymphoma-extra large; BET, Bromodomain and extraterminal domain protein, BETi, BET inhibitor; BM, Bone marrow; BRCA1, Breast cancer type 1 susceptibility protein; BRCA2, Breast cancer type 2 susceptibility protein; BRD4, Bromodomain-containing protein 4; CALR, Calreticulin; CD123, Cluster of differentiation 123; CD123i, CD123 inhibitor; CD131, Cluster of differentiation 131; CDK4, Cyclin-dependent kinase 4; CDK6, Cyclin-dependent kinase 6; CDK4/6i, CDK4/6 inhibitor; CIP2A, Cancerous inhibitor of PP2A; D-NHEJ, DNA-dependent protein kinase catalytic subunit-mediated non-homologous end-joining; DNA, Deoxyribonucleic acid; DSBs, Double-strand breaks; E2F1, E2F transcription factor 1; EF2, Elongation factor 2; ERp57, Endoplasmic reticulum resident protein 57; FGF-2, Fibroblast growth factor-2; HAT, Histone acetyltransferases; HDAC, Histone deacetylases; HDACi, HDAC inhibitor; HSP27, Heat shock protein 27; HSP27i, HSP27 inhibitor; HSP90, Heat shock protein 90; HSP90i, HSP90 inhibitor; hTR, Human telomerase RNA; IFNα, Interferon-alpha; IKK, IκB kinase; IL-1, Interleukin-1; IL-3, Interleukin-3; IL-6, Interleukin-6; IL-8, Interleukin-8; IκBα , Inhibitor of NF-κB, alpha; JAK, Janus kinase; JAK1, Janus kinase 1; JAK1/2i; JAK1/2 inhibitor; JAK2, Janus kinase 2; JAKi, JAK inhibitor; LKB1, Liver kinase B1; LSD-1, Lysine-specific histone demethylase 1; LSD-1i, LSD-1 inhibitor; MAX, Myc-associated factor X; Mcl-1, Induced myeloid leukaemia cell differentiation protein Mcl-1; MDM2, Mouse double minute 2 homolog; MDM2i, Mouse double minute 2 homolog inhibitor; me, Methyl-group; MHC-I, Major histocompatibility complex I; miR 375, microRNA 375; MPL, Promyelocytic leukaemia protein; MPN, Myeloproliferative neoplasm; mTORC1, Mammalian target of rapamycin complex 1; mTORC2, Mammalian target of rapamycin complex 2; Mut CALR, Mutant calreticulin; Myc, Myelocytomatosis oncogene; NF-κB, Nuclear factor kappa-light-chain-enhancer of activated B cells; NPM, Nucleophosmin; p27, Cyclin-dependent kinase inhibitor p27; p38, p38 mitogen-activated protein kinases; p50, p50 subunit of NF-κB; p53, p53 upregulated modulator of apoptosis; p65, p65 subunit of NF-κB; Pan-PI3Ki, Pan-PI3K inhibitor; PARP, Poly (ADP-ribose) polymerase; PARPi, PARP inhibitor; PDGF, Platelet derived growth factor; PDK1, 3-phosphoinositide-dependent protein kinase-1; Peg-IFNα-2a, Pegylated interferon alfa-2a; PI3K, Phosphoinositide 3-kinase; PI3K-delta I, PI3K-delta inhibitor; PI3K/mTORi, PI3K/mTOR inhibitor; PI3Ki, PI3K inhibitor; PIM, Proviral integration site for Moloney murine leukaemia virus kinase; PIMi, PIM inhibitor; PIP3, phosphatidylinositol-3, 4, 5-triphosphate; PMF, Primary myelofibrosis; PML, Promyelocytic leukaemia; PML-NB, Promyelocytic leukaemia-nuclear body; Ras, Rat sarcoma viral oncogene; PP2A, Protein phosphatase 2A; PUMA, p53 upregulated modulator of apoptosis; Rb, Retinoblastoma protein; Rheb, Ras homolog enriched in brain; ROS, Reactive oxygen species; S6K1, Ribosomal protein S6 kinase beta-1; SNAI1, Snail Family Transcriptional Repressor 1; STAT3, Signal transducer and activator of transcription 3; STAT5, Signal transducer and activator of transcription 5; TAP, Transporter associated with antigen processing;

TAPBP, Tapasin; Telomerase i, Telomerase inhibitor; TNFα, Tumour necrosis factor alpha; TPO, Thrombopoietin; TSC1, Tuberous sclerosis complex 1; TSC2, Tuberous sclerosis complex 2; TYK2, Tyrosine kinase 2; VGEF, Vascular endothelial growth factor; XPO1, Exportin 1; XPO1i, Exportin 1 inhibitor

Supplemental file 6. The bone marrow microenvironment in Philadelphia chromosome-negative myeloproliferative neoplasms and its therapeutic targeting.
Abbreviations: ActRIIA-Fc, Activin receptor type IIA- fragment crystallizable domain of human IgG1 antibody; ALK5, Activin receptor-like kinase 5; AURKA, Aurora kinase; CALR, Calreticulin; CAR Cells, CXCL12-abundant reticular cells; CD41, Cluster of differentiation 41; CD42, Cluster of differentiation 42; CXCL12, C-X-C Motif Chemokine Ligand 12; CXCR4, C-X-C Motif Chemokine Ligand 4; GATA1, GATA-binding factor 1; GDF11, Growth differentiation factor 11; HSC, Hematopoietic stem cell; IL-1β, Interleukin-1β; IL-8, Interleukin-8; JAK, Janus kinase; JAK2, Janus kinase 2; LCN2, Lipocalin-2; LOX2, Lipoxygenase 2; MMP3, Matrix metalloproteinase-3;MPL, Myeloproliferative leukaemia protein; MPN, Myeloproliferative neoplasms; MSC, Mesenchymal stromal cells; Myc, Myelocytomatosis oncogene; NE, Noradrenaline; SAP, Serum amyloid P component; SDF1/, Stromal cell-derived factor 1; SMAD 4, Mothers against decapentaplegic homolog 4; SMAD2/3, Mothers against decapentaplegic homolog 2/3; SNS, Sympathetic nervous system; Sp1, specificity protein 1; STAT, Signal transducer and activator of transcription; TGF, Transforming growth factor beta; TGFR1, TGF receptor 1; TGFR2, TGF receptor 2; TIMP1, TIMP metallopeptidase inhibitor 1; β3-AR, β3 adrenergic Receptor

Supplemental file 7. Immunologic pathways and their therapeutic targeting in Philadelphia chromosome-negative myeloproliferative neoplasms.
Abbreviations: CALR, Calreticulin; CTL, Cytotoxic T lymphocyte; ERK, Extracellular-signal-regulated kinase; IL-10, Interleukin-10; LSC, Leukemic stem cell; MDSC, Myeloid derived suppressor cells; MEK, Mitogen-activated protein kinase kinase; MHC-II, Major histocompatibility complex II; MPN, Myeloproliferative neoplasm; Mut CALR, Mutant calreticulin; PD-1, Programmed cell death protein 1; PD-L1, Programmed cell death ligand 1; STAT, Signal transducer and activator of transcription; STAT3, Signal transducer and activator of transcription 3; TLR-2, Toll-like receptor 2; Treg, Regulatory T cell
